# Repurposing FDA-Approved Drugs for Eumycetoma Treatment: Homology Modeling and Computational Screening of CYP51 Inhibitors

**DOI:** 10.3390/ijms26010315

**Published:** 2025-01-01

**Authors:** Magdi Awadalla Mohamed, Mohamed Khalid Alhaj Awadalla, Malik Suliman Mohamed, Tilal Elsaman, Eyman Mohamed Eltayib

**Affiliations:** 1 Department of Pharmaceutical Chemistry, College of Pharmacy, Jouf University, Sakaka 72388, Saudi Arabia; 2 Pharmacy Program, Wad Medani College of Medical Sciences and Technology, Wad Medani 21111, Gezira, Sudan; mkaawadalla@yahoo.com; 3 Department of Pharmaceutics, College of Pharmacy, Jouf University, Sakaka 72388, Saudi Arabia; msmustafa@ju.edu.sa (M.S.M.); emeahmed@ju.edu.sa (E.M.E.)

**Keywords:** eumycetoma, drug discovery, repurposing, CYP51, homology modeling

## Abstract

Eumycetoma, a chronic fungal infection caused by *Madurella mycetomatis*, is a neglected tropical disease characterized by tumor-like growths that can lead to permanent disability and deformities if untreated. Predominantly affecting regions in Africa, South America, and Asia, it imposes significant physical, social, and economic burdens. Current treatments, including antifungal drugs like itraconazole, often show variable efficacy, with severe cases necessitating surgical intervention or amputation. Drug discovery for eumycetoma faces challenges due to limited understanding of the disease’s molecular mechanisms and the lack of 3D structures for key targets such as *Madurella mycetomatis* CYP51, a well-known target for azoles’ antifungal agents. To address these challenges, this study employed computational approaches, including homology modeling, virtual screening, free energy calculations, and molecular dynamics simulations, to repurpose FDA-approved drugs as potential treatments for eumycetoma targeting *Madurella mycetomatis* CYP51. To this end, a library of 2619 FDA-approved drugs was screened, identifying three promising candidates: montelukast, vilanterol, and lidoflazine. These compounds demonstrated favorable binding affinities, strong interactions with critical residues of the homology model of *Madurella mycetomatis* CYP51, and stability in molecular dynamics simulations, offering potential for further investigation as effective therapeutic options for eumycetoma.

## 1. Introduction

Mycetoma, also known as Madura foot, is a chronic, granulomatous, soft tissue infection caused by either bacteria (actinomycetoma) or fungi (eumycetoma) [1,2,3]. Eumycetoma, is not limited to a specific geographic area; it is a broader global health concern that can affect individuals in various regions around the world. However, it is more prevalent in rural, agricultural areas within tropical and subtropical regions, particularly in what is known as the “Mycetoma belt”, spanning parts of Africa, Latin America, and Asia [4]. Eumycetoma is generally acquired through traumatic inoculation, where a sharp, contaminated object penetrates the skin, introducing the causative organism, most commonly *Madurella mycetomatis* [5]. The infection typically affects the feet, hands, or lower legs and progresses gradually, leading to the classical triad of a painless, indurated subcutaneous mass, multiple discharging sinuses, and the presence of grains in the purulent or seropurulent discharge. The mass, which feels woody and hard, gradually enlarges over time, while the sinus tracts drain fluid containing fungal granules of varying colors, depending on the causative organism. If left untreated, the infection can extend to deeper tissues and bones, causing deformities, disability, and potentially death. This progression and the characteristic features of the disease are crucial for its diagnosis and for distinguishing it from other chronic subcutaneous infections [1,2,3,4,5,6,7,8,9,10,11,12].

Treatment of eumycetoma is particularly challenging due to several factors, including the chronic nature of the disease, poor drug penetration into fibrotic tissue, and the limited efficacy, significant toxicity, and high cost of currently used antifungal therapies, along with diagnostic delays in resource-limited endemic areas [2]. Additionally, surgical intervention, which is often necessary, is complex and carries a high risk of recurrence [4,6]. Currently, no universally effective treatment exists [13,14], highlighting the urgent need for more accessible and effective therapeutic strategies.

As a neglected tropical disease [1], eumycetoma suffers from a severe lack of attention from research and development companies, compounded by economic and clinical barriers. To date, not a single druggable target for eumycetoma has been resolved as an X-ray crystal structure, highlighting the critical need for innovative approaches to address this disease [15,16,17,18,19,20,21,22,23,24,25,26,27]. These challenges—spanning clinical, economic, and research-related issues—have severely hindered progress in drug discovery and development, further complicating the management of the disease. To address this pressing issue, the current study constructed a homology model of *Madurella mycetomatis* CYP51 and screened it against a library of FDA-approved drugs to identify potential candidates for repurposing as eumycetoma treatments.

## 2. Results and Discussion

### 2.1. Challenges and Perspectives in Drug Discovery for Eumycetoma

Despite the severity of eumycetoma, therapeutic options remain limited in efficacy, often resulting in significant side effects, incomplete disease resolution, or recurrence. Various classes of antifungals have been employed, with azoles being the most commonly used [28]. Ketoconazole was the drug of choice until 2013 when it was withdrawn by the FDA due to severe hepatic and adrenal toxicity [18,29,30]. Currently, itraconazole is the primary treatment option; however, its outcomes are frequently suboptimal, even after prolonged treatment periods, which carry the risk of hepatotoxicity [2]. Other antifungal agents, such as terbinafine and liposomal amphotericin B, have also been used, but their success has been limited, largely due to poor drug penetration. Factors such as the fibrous nature of the lesion, the presence of melanin in fungal grains, and excessive collagen encapsulation of *Madurella mycetomatis* contribute to treatment failure [2,4,15,18,19,20,21,22,23,24,25,26,27,31,32]. Moreover, the high cost of antifungal therapy imposes a severe financial burden on patients in endemic areas, compounded by the risk of toxicity from prolonged treatment [2,7,28,33]. Furthermore, in advanced cases of eumycetoma where the infection has invaded deeper tissues and bones, surgical intervention is necessary to improve the treatment outcomes of itraconazole [6,7,9,16,17,21,34,35]. However, this treatment plan has limited success, with low cure rates and a high risk of recurrence [2,36]. Along with these limitations, early and late complications associated with surgical intervention are among the major drawbacks of this treatment plan [37]. Thus, there is an urgent need to find new cost-effective medications for the treatment of eumycetoma. Yet, as a neglected tropical disease, eumycetoma receives little attention from the pharmaceutical industry due to the high costs and low financial returns associated with drug development [1,38,39,40]. As a result, drug discovery for this disease is slow and challenging, further hindered by the limited characterization of drug targets despite the availability of the *Madurella mycetomatis* genome sequence since 2016 [41]. Hence, drug discovery efforts are predominantly led by universities, specialized research institutes, and dedicated initiatives. Even so, these studies often rely on broad, non-rational methods that are both costly and time-consuming [42,43,44]. Therefore, drug repurposing has emerged as a promising strategy for finding effective treatments for eumycetoma [45].

**Figure 1:**
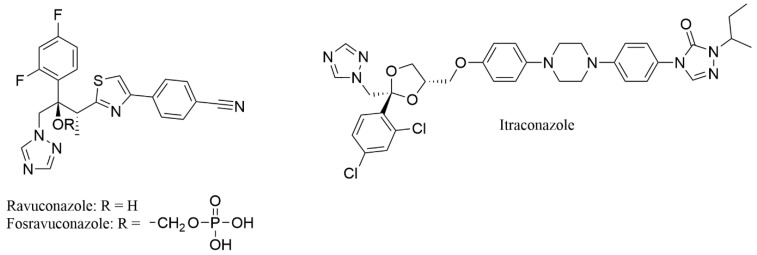
Chemical structures of azole antifungals. Itraconazole is the current treatment for eumycetoma [2,36]. Ravuconazole is the active form of the prodrug fosravuconazole which has been clinically assessed for its potential as a new treatment for eumycetoma [36].

Drug repurposing, or drug repositioning, involves identifying new therapeutic applications for existing medications [46]. This strategy leverages drugs already approved, enabling a faster development timeline as these compounds have been tested for human safety. Accordingly, for eumycetoma, repurposing FDA-approved drugs could significantly reduce the time and cost typically associated with drug development, as these compounds have already demonstrated safety and efficacy through rigorous testing [47,48]. In this context, azoles, a class of antifungal agents, are particularly promising, as they work by inhibiting CYP51, a key enzyme in fungal sterol biosynthesis, thereby disrupting fungal cell membrane synthesis. Specifically, fosravuconazole (Figure 1), an oral water-soluble prodrug that rapidly metabolizes into ravuconazole, was initially developed for onychomycosis and has demonstrated promising efficacy against eumycetoma [9]. To explore its potential for treating eumycetoma, a phase 2 clinical trial was conducted at the Mycetoma Research Centre in Sudan, beginning in 2017 and concluding in 2021 [36]. This randomized, double-blind, active-controlled, superiority study aimed to compare the efficacy of two dosing regimens of fosravuconazole with the standard treatment, itraconazole, both administered in conjunction with surgery. The results indicated that neither dose of fosravuconazole outperformed itraconazole, and both doses showed slightly lower efficacy. Despite this, fosravuconazole raised no new safety concerns and offered advantages, such as a reduced pill burden and fewer drug-drug interactions, compared to the more costly and less accessible itraconazole. These findings support the need for continued research into more effective treatments with shorter durations and higher cure rates, ideally reducing the reliance on surgery.

Drug discovery involves a series of steps: identifying and validating drug targets, discovering and optimizing leads, and conducting preclinical and clinical studies [49]. In silico drug design has become integral to this process, particularly in lead identification and optimization as it enhances understanding of target–ligand binding interactions [50,51]. It includes structure-based (direct) and ligand-based (indirect) approaches, both extensively applied in exploring potential leads [52]. Molecular docking is the most widely used in silico structure-based drug design approach, with a history spanning over 40 years [53,54]. It allows atomic-level modeling of ligand–target interactions, predicting ligand conformation, position, and orientation within the target’s active site [55]. Therefore, molecular docking enables efficient computational screening of millions of ligands, reducing the time and cost of lead identification [56,57,58].

The 3D structure of a protein is crucial in molecular docking as it reveals the shape, size, and specific arrangement of amino acids within the binding site, allowing for accurate prediction of how well a ligand, like a drug, can fit and bind [54]. This spatial detail enables docking software to assess key interactions (hydrogen bonds, hydrophobic contacts, and electrostatics) and calculate binding affinities, essential for predicting drug effectiveness [59,60]. Additionally, the 3D structure captures the protein’s flexibility, which is important for understanding potential conformational changes upon ligand binding, leading to more reliable docking outcomes [61]. X-ray crystallography is the most preferred experimental technique for the 3D structure determination of proteins and other biological membranes [62]. On the other hand, homology modeling is a computational method that predicts a protein’s 3D structure based on its amino acid sequence, using known structures of similar proteins. It is widely regarded as one of the most accurate approaches for structural prediction among computational techniques. This method enables the creation of reliable structural models, even when experimental data for the protein of interest is unavailable [63].

As of now, to the best of our knowledge, not a single rational drug discovery for eumycetoma has been fully reported [64]. This could be partly due to the limited knowledge of specific molecular targets identified for eumycetoma-causing fungi [2]. The cytochrome P450 enzyme lanosterol 14α-demethylase (CYP51) is crucial for the biosynthesis of ergosterol, a key component of fungal cell membranes that regulates their fluidity, permeability, and structure. Inhibiting CYP51 disrupts ergosterol production, leading to impaired fungal cell growth, making this enzyme a recognized target for antifungal drugs such as azoles [65,66,67,68,69,70,71,72,73,74]. Interestingly, the CYP51 enzyme of *Madurella mycetomatis* has been identified [41]. However, the absence of its three-dimensional structure has hindered the design of novel inhibitors for the treatment of eumycetoma, despite the enzyme’s critical role. To address the challenge of developing new therapeutic agents for eumycetoma, this study constructed a homology model of *Madurella mycetomatis* CYP51 using a template with the highest amino acid sequence similarity to the target protein. The model, built using YASARA (Yet Another Scientific Artificial Reality Application) protein modeling program [75], was employed to screen FDA-approved drugs [76] for potential repurposing, with itraconazole, the current standard treatment for eumycetoma, serving as the reference ligand (Figure 1).

### 2.2. Homology Modeling and Validation

**Figure 2:**
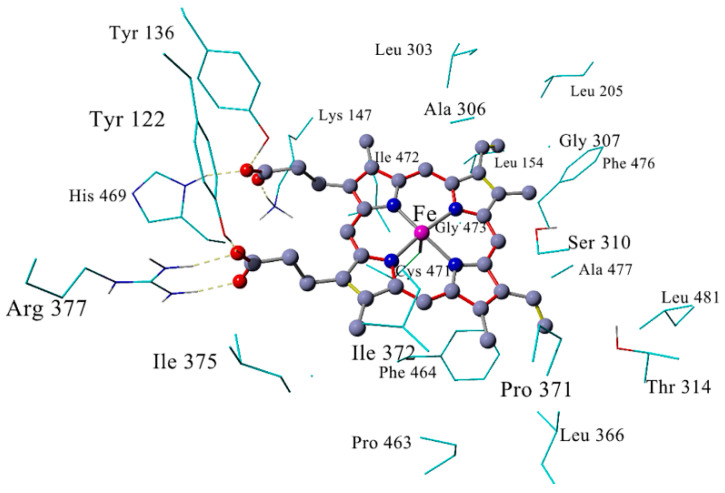
The active site of the *Madurella mycetomatis* CYP51 homology model. The heme group is illustrated in ball and stick representation, while crucial residues engaged in hydrophobic interactions with the heme are displayed in the stick model. Hydrogen bonds are depicted as yellow dashed lines. In the heme group, single and double bonds are indicated by grey and yellow lines, respectively. Resonance bonds are represented by red lines, and iron coordination bonds are depicted by light grey lines. To maintain clarity, only the side chains of amino acids are presented.

The primary structure of the *Madurella mycetomatis* CYP51 enzyme, consisting of 529 amino acids (available at: https://www.ncbi.nlm.nih.gov/protein/KXX80456, accessed on 1 March 2023), was obtained from the first genome sequence of *Madurella mycetomatis*, with the locus tag “MMYC01_202883”, which was isolated from a human eumycetoma case in Sudan [41]. A BLASTp search conducted within the experimentally determined Protein Data Bank (PDB) (www.rcsb.org, accessed on 1 March 2023)) structures revealed an X-ray solved sterol 14α-demethylase CYP51B from *Aspergillus fumigatus* (PDB code 6CR2) [77] as the template with the highest sequence identity (64.71%), lowest E-value (0.0), a coverage of (89%) and similar Max and Total scores (665). The pairwise alignment between *Madurella mycetomatis* CYP51 (Query) and *Aspergillus fumigatus* CYP51B (Subject) sequences, generated using NCBI BLASTp, is shown in Supplementary Figure S1. Therefore, the *Aspergillus fumigatus* CYP51B structure coordinate file in PDB format (PDB ID: 6CR2) was used as a template to build the target homology model, employing the FASTA format of *Madurella mycetomatis* CYP51 amino acid sequence (accession number: KXX80456). The model was constructed using the YASARA protein modeling program [75]. Analysis of hydrophobic interactions within a 5 Å cutoff distance revealed numerous interactions between the model residues and the heme group, particularly with the active site residues Leu154, Leu205, Leu303, Ala306, Gly307, Ser310, Thr314, Leu366, Pro371, Ile372, Ile375, Pro463, Phe464, Ile472, Gly473, Phe476, Ala477, and Leu481. Hydrogen bonds were observed between the heme carboxyl groups and the side chains of Tyr122, Tyr136, Lys147, Arg377, and His469. Furthermore, the sulfide group of Cys471 occupied the fifth coordination position of the heme iron atom. These interactions collectively facilitated the precise positioning of the heme group within the model active site (Figure 2).

Assessment of the dihedral angles of the model residues conducted via the MolProbity server (http://molprobity.biochem.duke.edu, accessed on 2 March 2023) revealed that 97.1% of the residues reside within the favored regions of the Ramachandran plot. Additionally, 13 residues (2.7%) are located within the allowed regions, while only one residue Leu125 (0.2%) is situated in the outlier, area similar to Ramachandran analysis of the template (Supplementary Figure S2a,b). This outcome suggests that the stereochemical accuracy of the model is comparable to that of X-ray-solved CYP51B from *Aspergillus fumigatus* (PDB: 6CR2). Verify3D (https://www.doe-mbi.ucla.edu, accessed on 5 March 2023) evaluates the compatibility between a protein’s atomic model (3D) and its amino acid sequence (1D). It assigns a structural class based on the environment and location of each residue (e.g., alpha, beta, loop, polar, and nonpolar) and compares these results to those of well-refined structures. This comparison helps distinguish correct protein folding from errors, ensuring the model’s structural accuracy. In this context, 85.69% of the model residues scored ≥ 0.1 in the Verify3D-1D profile, exceeding the 80% threshold for “passing” the assessment, indicating that the model has an acceptable folding reliability (Supplementary Figure S3). The QMEAN server (https://swissmodel.expasy.org/qmean/, accessed on 9 March 2023) evaluates a model absolute quality by combining a linear combination of statistical potential terms and comparing it to scores from a non-redundant set of high-resolution X-ray crystallographic structures. The *Madurella mycetomatis* CYP51 model normalized QMEAN z-score felt within the standard deviation value often reported for native proteins of similar size (Supplementary Figure S4), indicating that the model’s absolute quality is equivalent to that of previously solved structures. These findings clearly demonstrate the reliability and validity of the constructed homology model of *Madurella mycetomatis* CYP51, paving the way for future computational investigations aimed at repurposing FDA-approved drugs for the treatment of eumycetoma.

### 2.3. Virtual Screening and MM-GBSA Binding Free Energy Calculations

The repurposing of FDA-approved drugs for eumycetoma treatment should focus on both molecular and practical aspects to determine efficacy and suitability. Molecular criteria, such as docking scores and MM-GBSA energies, assess each drug’s binding affinity to CYP51, emphasizing interactions with key residues in the enzyme’s active site. Lipophilicity, solubility, and ADMET properties further ensure bioavailability and safety, critical for treating eumycetoma in low-resource settings. Practical considerations, including affordability, scalability, and minimal resistance risk, are also prioritized to make treatments viable and accessible. Ethical and regulatory aspects ensure that drug repurposing does not interfere with availability for the original indications. Following this approach would help propose the most promising candidates for further testing and clinical trials to treat eumycetoma effectively.

Having built the homology model, the study next focused on analyzing the interaction of itraconazole, the first-line treatment of eumycetoma [2,36], with *Madurella mycetomatis* CYP51. LigPrep (Schrödinger Release 2023-2: LigPrep, Schrödinger, LLC, New York, NY, USA, 2023) generated two ionization states of itraconazole: (a) the unionized form and (b) the ionized form. Docking results revealed scores of −5.115 kcal/mol for the ionized form and −7.049 kcal/mol for the unionized form (Table 1).

**Figure 3:**
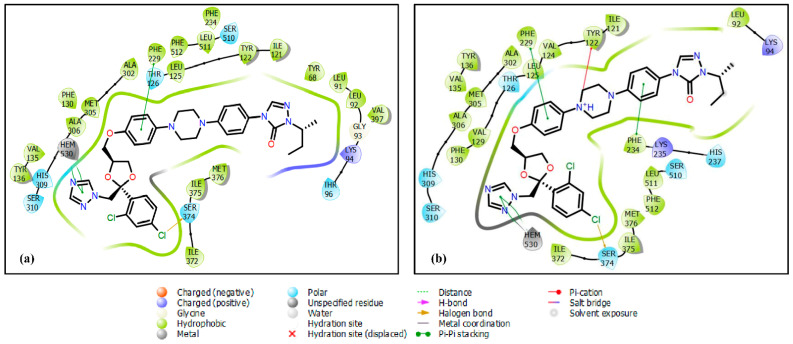
The 2D interactions of itraconazole’s (**a**) unionized form and (**b**) ionized form with the homology model of the *Madurella mycetomatis* CYP51 binding site.

The unionized itraconazole exhibited notable binding interactions, including two Pi–Pi stacking interactions with the heme group at distances of 4.67 Å and 5.35 Å. Additionally, it formed a halogen bond with Ser374 (3.14 Å) and a Pi–Pi stacking interaction with Phe229 (5.00 Å). Hydrophobic interactions were also observed between the unionized itraconazole and multiple residues within the CYP51 binding site. These included Tyr68, leu91, leu92, Ile121, Tyr122, Leu125, Phe130, Val135, Tyr136, Phe234, Ala302, Met305, Ala306, Ile372, Ile375, Met376, Leu511, and Phe512, contributing significantly to the drug’s binding affinity and stabilization within the active site. On the other hand, the ionized form of itraconazole demonstrated distinct binding interactions within the CYP51 active site. It engaged in a Pi–cation interaction with Tyr122 (5.84 Å), Pi–Pi stacking interactions with Phe229 (4.73 Å) and Phe234 (4.13 Å), a halogen bond with Ser374 (3.18 Å), and Pi–Pi stacking interactions with the heme group at 4.27 Å and 4.76 Å. Additionally, hydrophobic interactions were observed with residues Leu92, Ile121, Val124, Leu125, Val129, Phe130, Val135, Tyr136, Ala302, Met305, Ala306, Ile372, Ile375, Met376, Leu511, and Phe512, further stabilizing the binding (Figure 3).

The in-house homology model of *Madurella mycetomatis* CYP51 was used to screen a library of 2619 FDA-approved drugs retrieved from the DrugBank database (https://go.drugbank.com/, accessed on 25 March 2023) [76] by utilizing the virtual screening workflow within the Maestro graphical interface in Schrödinger (Schrödinger Release 2023-2: Maestro, Schrödinger, LLC., New York, NY, USA, 2023). This virtual screening workflow applied several core tools from the Schrödinger suite, including Protein Preparation Wizard [78], LigPrep (Schrödinger Release 2023-2: LigPrep, Schrödinger, LLC., New York, NY, USA, 2023), Glide [79], and Prime [80], to systematically identify potential drugs targeting *Madurella mycetomatis* CYP51. Virtual screening commenced with high-throughput virtual screening (HTVS) to filter out low-affinity compounds, followed by standard precision (SP) and extra precision (XP) docking for a more detailed analysis. Finally, MM-GBSA calculations were conducted to evaluate binding energies, confirming the stability of selected ligands and narrowing down the pool to promising candidates for experimental validation [81].

The final docking scores from the XP step identified 20 top drugs, with diosmin (−13.919 kcal/mol) and montelukast (−13.813 kcal/mol) showing the strongest predicted affinities for *Madurella mycetomatis* CYP51, indicating highly favorable interactions within the binding pocket. Although latanoprostene bunod had the weakest docking score among the screened drugs (−8.879 kcal/mol), it still demonstrated a better fit than itraconazole (−5.115 kcal/mol and −7.049 kcal/mol), the current standard treatment for eumycetoma (Table 1). These results suggest that all 20 top-ranked drugs, despite varying docking scores, have potential for further exploration as candidates for eumycetoma treatment. Docking scores, while useful for predicting initial binding affinities, can lead to false positives due to their inability to fully account for dynamic receptor behavior, solvation, and entropy [47,49,82]. Therefore, further validation through methods like MM-GBSA binding energy calculations or molecular dynamics (MD) simulations is crucial to confirm the stability and reliability of these interactions [81]. For instance, docking results suggest that the unionized form of itraconazole has a slightly better initial binding affinity (−7.049 kcal/mol) compared to the ionized form (−5.115 kcal/mol). However, MM-GBSA results revealed that the ionized form is significantly more stable and energetically favorable, with a binding affinity of −47.19 kcal/mol versus −35.18 kcal/mol for the unionized form (Table 1). This highlights the necessity of complementing docking studies with post-docking analyses like MM-GBSA, which offers a more comprehensive evaluation by considering solvation, receptor flexibility, and entropic effects. Such an approach mitigates the risk of over-reliance on docking scores, which may occasionally misrepresent the true binding potential of drug candidates.

The MM-GBSA results for various drugs docked against *Madurella mycetomatis* CYP51 revealed varying degrees of binding affinity (Table 1). Bemotrizinol, a sunscreen [76], displayed the best MM-GBSA score of −82.13 kcal/mol, indicating the strongest binding interaction among the drugs tested. This was followed by vilanterol (−66.69 kcal/mol) and lidoflazine (−62.76 kcal/mol), which also showed relatively strong interactions. Imatinib, montelukast, and latanoprostene bunod demonstrated moderate binding affinities, with scores slightly higher than itraconazole (−49.90 kcal/mol, −48.82 kcal/mol, −48.15 kcal/mol, and −47.19 kcal/mol, respectively).

Sunscreens are designed for topical application, with minimal absorption into the bloodstream, which restricts their ability to reach internal infection sites and meet the pharmacokinetic criteria required for systemic use [83]. Repurposing sunscreens would necessitate significant modifications and rigorous clinical testing to assess their safety and efficacy in new therapeutic indications, such as eumycetoma treatment. Therefore, despite bemotrizinol’s potential binding affinity, it was excluded from further analysis for repurposing in the treatment of eumycetoma. Although imatinib exhibits a binding affinity comparable to itraconazole (Table 1), it is unsuitable for repurposing as a treatment for eumycetoma due to its primary role as an anticancer agent [76]. Its use in a non-oncological context, such as treating eumycetoma, could pose unforeseen risks, necessitating extensive evaluation. This process would require lengthy regulatory approvals, including new safety and efficacy studies, significantly increasing costs and delaying access. Moreover, anticancer drugs are typically expensive due to their complex development and manufacturing processes, rendering them financially inaccessible for eumycetoma patients, especially in low-income, endemic regions [84]. As a result, imatinib was excluded from further analysis for repurposing in the treatment of eumycetoma. Latanoprostene bunod, primarily used as an ocular hypotensive agent for treating glaucoma and ocular hypertension, faces significant challenges for repurposing in eumycetoma treatment [76,85]. Designed for topical ocular use, its systemic absorption is minimal, making it unlikely to achieve therapeutic levels in the bloodstream necessary for targeting eumycetoma. Furthermore, the drug’s safety profile is optimized for ophthalmic application and has not been evaluated for systemic use, raising concerns about potential toxicity and unforeseen risks in a non-ocular context. Accordingly, repurposing latanoprostene bunod for systemic treatment would require substantial modifications to its formulation, along with extensive clinical trials to establish its safety and efficacy for this new indication. These steps would entail significant regulatory hurdles and high costs, making the process complex and challenging. As a result, latanoprostene bunod was excluded from further consideration for repurposing as a treatment for eumycetoma. In light of the above and based on the MM-GBSA analysis (Table 1), vilanterol, lidoflazine, and montelukast emerged as promising candidates for further analysis due to their promising binding affinity with the homology model of *Madurella mycetomatis* CYP51, displaying better affinity than itraconazole. Moreover, they are clinically used for systemic treatment and are available in oral formulations, which is advantageous for accessibility and patient compliance [76,86].

Vilanterol, a long-acting β2-adrenergic receptor agonist, is used in respiratory conditions like asthma and chronic obstructive pulmonary disease (COPD), with potential systemic anti-inflammatory effects [87]. Lidoflazine, a calcium channel blocker with anti-ischemic properties, has demonstrated efficacy in systemic cardiovascular conditions [88]. Montelukast, a leukotriene receptor antagonist, is widely prescribed for asthma and allergic rhinitis, offering systemic anti-inflammatory benefits alongside excellent oral bioavailability and affordability [89]. These attributes, coupled with their promising interactions with the fungal CYP51 enzyme, make them strong candidates for further exploration in the context of eumycetoma treatment.

Azoles are antifungal agents that inhibit CYP51, disrupting fungal cell membrane synthesis. Due to their targeted action against fungal infections and known safety profiles, they are strong candidates for repurposing, particularly against *Madurella mycetomatis* CYP51. As mentioned earlier, fosravuconazole (Figure 1) has been explored in clinical trials for treating eumycetoma, with itraconazole as the comparator drug. Despite its promising potential, clinical trial results revealed that treatment with fosravuconazole did not show superior efficacy compared to itraconazole, suggesting that fosravuconazole may not offer a significant advantage over the existing treatment [36]. In this study, ravuconazole, the active form of fosravuconazole, was tested for its binding affinity to *Madurella mycetomatis* CYP51 using MM-GBSA calculations. The binding energy of ravuconazole (−31.85 kcal/mol) was weaker compared to itraconazole (−47.19 kcal/mol), indicating that itraconazole has a stronger predicted interaction with the enzyme (Table 1). This difference in binding affinity may partly explain the clinical trial findings, where fosravuconazole, the prodrug of ravuconazole, did not show superior efficacy compared to itraconazole [36].

### 2.4. Molecular Dynamics (MD) Simulations

With the complexes of vilanterol, lidoflazine, and montelukast bound to the homology model of *Madurella mycetomatis* CYP51 established, the study turned to evaluating the structural changes in the protein. This analysis was conducted using RMSD, RMSF, hydrogen bonding, and hydrophobic interactions to gain insights into the dynamics and stability of the complexes [90]. In this regard, MD simulations of the *Madurella mycetomatis* CYP51 homology model, both in its apo form and in complexes with the selected drugs, itraconazole, ravuconazole, vilanterol, montelukast, and lidoflazine, revealed distinct interaction profiles and varying stability, highlighting differences in dynamic behavior between the bound and unbound states.

**Figure 4:**
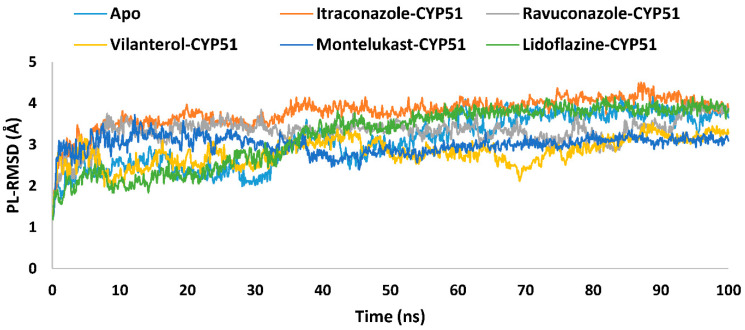
The PL-RMSD analysis of the apoprotein of the *Madurella mycetomatis* CYP51 homology model and its complexes with itraconazole, ravuconazole, vilanterol, montelukast, and lidoflazine monitored during the MD simulations trajectories.

Using itraconazole as a reference, Protein–Ligand Root Mean Square Deviation (PL-RMSD) analysis identified variations in stability among the drugs (Figure 4). PL-RMSD reflects the conformational stability of the complex by monitoring the Cα-RMSD of all atoms throughout the simulation. The stability of the complex is inversely correlated with PL-RMSD variation: lower RMSD variation indicates greater stability [49]. The apo form demonstrated an average deviation of 3.1 Å, reflecting its inherent flexibility in the absence of a ligand. Among the drug-bound complexes, vilanterol exhibited the lowest average deviation (2.8 Å), indicating the most stable interaction, while itraconazole showed the highest average deviation (3.8 Å), indicating more significant induced conformational changes upon binding, with maximum deviations reaching 4.5 Å (Table 2).

Despite these variations, all PL-RMSD values were within acceptable ranges, confirming that the complexes maintained structural integrity throughout the simulation period [47,49,58,82].

**Figure 5:**
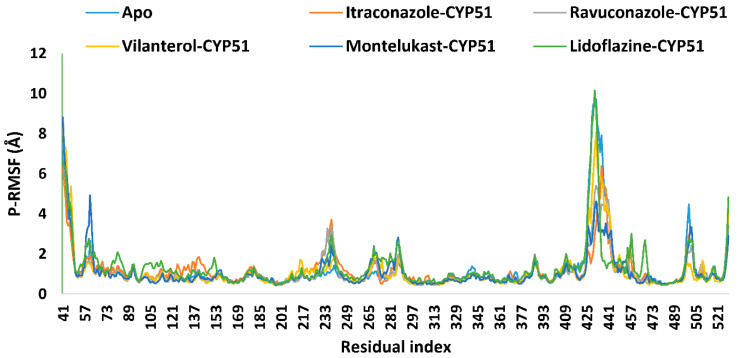
The P-RMSF analysis of the apoprotein of the *Madurella mycetomatis* CYP51 homology model and its complexes with itraconazole, ravuconazole, vilanterol, montelukast, and lidoflazine monitored during the MD simulations trajectories.

The Protein Root-Mean-Square Fluctuation (P-RMSF), which indicates residue-specific flexibility [47], revealed consistent structural stability across all CYP51-drug complexes, with average fluctuations ranging from 1.1 to 1.3 Å, comparable to the apo form’s average of 1.2 Å (Table 2 and Figure 5). This suggests that drug binding did not significantly impact overall stability. Notably, regions of greater flexibility were observed, with fluctuations reaching up to 10.2 Å in the lidoflazine complex. These higher fluctuations likely correspond to localized dynamic regions, such as loops or solvent-exposed areas, which are characteristically more mobile [49,91]. Moreover, this increased mobility in specific regions of the protein may result from structural rearrangements triggered by drug binding, which could facilitate or stabilize interactions within the binding site [92].

**Figure 6:**
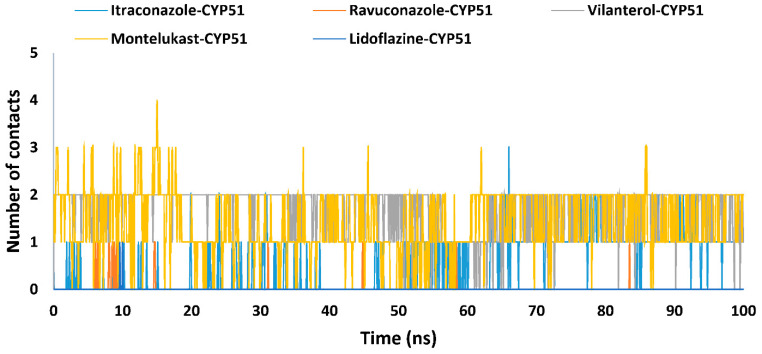
The number of H-Bonds contacts established during the entire MD simulations run for the complexes of the *Madurella mycetomatis* CYP51 homology model with itraconazole, ravuconazole, vilanterol, montelukast, and lidoflazine.

Hydrogen bonding and hydrophobic interactions play crucial roles in determining the stability and specificity of protein-ligand interactions [93]. These interactions varied significantly across the studied drug complexes, highlighting diverse binding mechanisms. The average number of hydrogen bond contacts per frame ranged from 0.0 in the lidoflazine-CYP51 and ravuconazole-CYP51 complexes to 1.7 in vilanterol’s. Montelukast stood out with the ability to form up to four hydrogen bonds, averaging 1.4, indicating strong anchoring within the CYP51 binding pocket. In contrast, itraconazole exhibited a lower average of 0.5 hydrogen bonds (Table 2 and Figure 6), suggesting that its binding stability depends more on hydrophobic interactions than on polar contacts, consistent with its efficacy as an antifungal [93,94].

**Figure 7:**
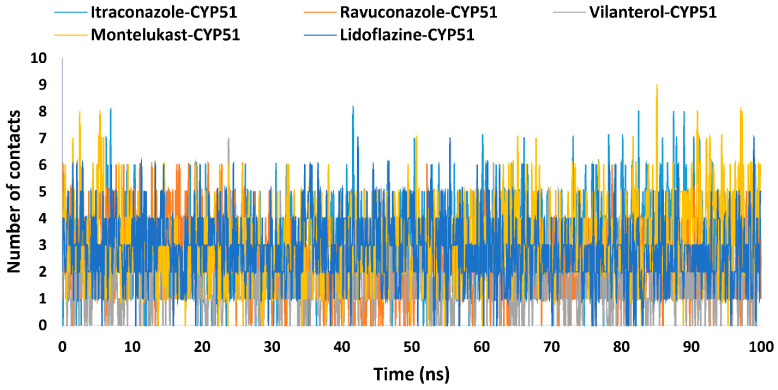
The number of hydrophobic contacts established during the entire MD simulations run for the complexes of the *Madurella mycetomatis* CYP51 homology model with itraconazole, ravuconazole, vilanterol, montelukast, and lidoflazine.

Hydrophobic interactions further highlighted the diversity in binding strategies (Table 2 and Figure 7). Montelukast showed the strongest engagement, averaging 3.2 contacts with a maximum of 9.0, reflecting its high affinity for nonpolar residues and strong overall stability. Lidoflazine exhibited comparable hydrophobic interactions, averaging 2.8 contacts, but its minimal hydrogen bonding suggests a predominantly nonpolar binding mechanism. Itraconazole displayed a balanced interaction profile, with moderate hydrophobic contacts (average 3.0) and occasional hydrogen bonds, peaking at 3.0 but averaging only 0.5. Vilanterol balanced fewer hydrophobic interactions (average 1.7) with consistent hydrogen bonding (average 1.7, peaking at 2.0), stabilizing its binding through a combination of polar and nonpolar interactions. Ravuconazole, on the other hand, maintained steady hydrophobic interactions (average 2.4) but exhibited minimal hydrogen bond formation (average 0.0, peaking at 1.0), emphasizing its preference for nonpolar binding dynamics [93,94]. Notably, montelukast stabilizes its binding through a combination of strong hydrophobic interactions and robust hydrogen bonding, whereas ravuconazole and lidoflazine primarily depend on nonpolar interactions. This interplay between hydrophobic and polar forces dictates the binding specificity and stability of drug candidates targeting CYP51, revealing a spectrum of binding mechanisms within the CYP51 binding pocket.

Montelukast’s strong hydrophobic interactions, particularly in the nonpolar pockets of the active site, suggest robust binding stability. Vilanterol’s hydrogen bonding, with an average of 1.7 contacts, highlights its potential for maintaining specificity and proper orientation within the binding pocket. These findings provide valuable insights into the binding dynamics of these drugs, emphasizing their potential as inhibitors of *Madurella mycetomatis* CYP51. In contrast, lidoflazine demonstrated relatively less stability when complexed with *Madurella mycetomatis* CYP51, as indicated by its relatively higher PL-RMSD and P-RMSF values (Table 2), moderate hydrophobic interactions (Table 2 and Figure 7), and weak hydrogen bonding within the enzyme’s binding pocket (Table 2 and Figure 6).

**Figure 8:**
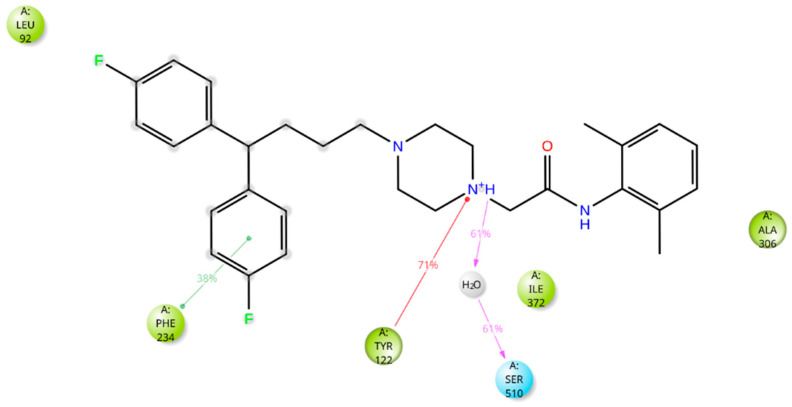
Lidoflazine’s interactions with the *Madurella mycetomatis* CYP51 homology model. Only interactions that occurred more than 30.0% of the simulation time in the selected trajectory (0 through 100 ns) are shown.

**Figure 9:**
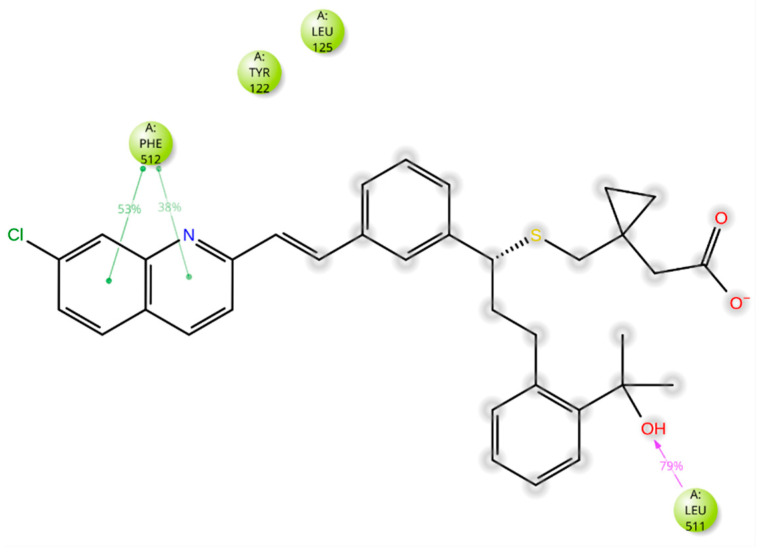
Montelukast’s interactions with the *Madurella mycetomatis* CYP51 homology model. Only interactions that occurred more than 30.0% of the simulation time in the selected trajectory (0 through 100 ns) are shown.

**Figure 10:**
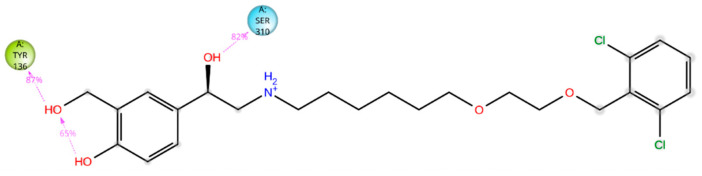
Vilanterol’s interactions with the *Madurella mycetomatis* CYP51 homology model. Only interactions that occurred more than 30.0% of the simulation time in the selected trajectory (0 through 100 ns) are shown.

**Figure 11:**
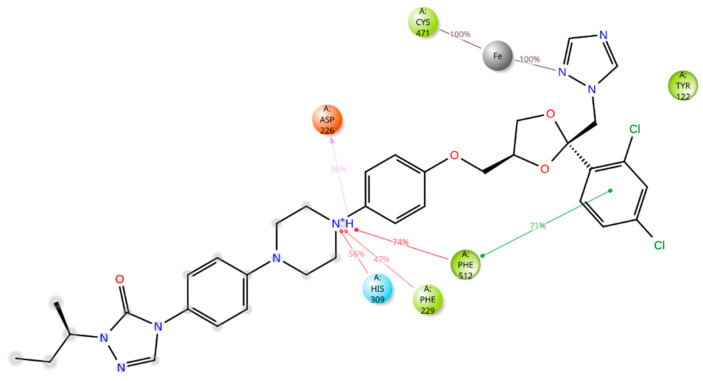
Itraconazole’s interactions with the *Madurella mycetomatis* CYP51 homology model. Only interactions that occurred more than 30.0% of the simulation time in the selected trajectory (0 through 100 ns) are shown.

**Figure 12:**
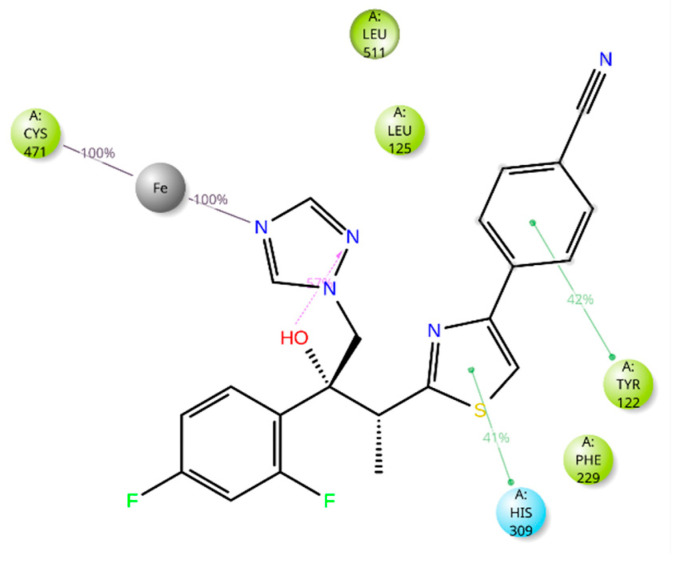
Ravuconazole’s interactions with the *Madurella mycetomatis* CYP51 homology model. Only interactions that occurred more than 30.0% of the simulation time in the selected trajectory (0 through 100 ns) are shown.

The five drugs consistently interacted with the enzyme’s binding pocket during the simulation, forming associations with various amino acids. However, these interactions were not uniform throughout the simulation. Some were stable and frequent, while others were intermittent or varied in strength. This indicates that the drugs’ binding to the enzyme was dynamic, with certain amino acids playing a more consistent role in the binding process and others contributing less consistently. Lidoflazine formed hydrophobic contacts with Leu92, Ala306, and Ile372 for more than 30% of the simulation time. Additionally, it exhibited Pi-Pi stacking with Phe234 (38%), Pi-cation interaction with Tyr122 (71%), and a water-mediated hydrogen bond with Ser510 (61%) (Figure 8 and Supplementary Figure S5). Montelukast displayed hydrophobic contacts with Tyr122 and Leu125 for over 30% of the simulation time, along with Pi-Pi stacking with Phe512 (53%) and a hydrogen bond with Leu511 (79%) (Figure 9 and Supplementary Figure S6). Vilanterol interacted with the CYP51 binding pocket via hydrogen bonds with Tyr136 and Ser310, which persisted for 87% and 82% of the simulation time, respectively (Figure 10 and Supplementary Figure S7). Itraconazole formed hydrophobic interactions with Tyr122 for over 30% of the simulation time, hydrogen bonding with Asp226 (36%) and Pi-cation interactions with His309 (56%), Phe229 (47%), and Phe512 (74%). Additionally, it displayed Pi-Pi stacking with Phe512 (71%). Throughout the simulation, itraconazole remained bound to the heme iron, which was also consistently bound to Cys471 (Figure 11 and Supplementary Figure S8). Ravuconazole exhibited hydrophobic interactions with Leu125, Phe229, and Leu511 for more than 30% of the simulation time. It demonstrated Pi-Pi stacking with Tyr122 (42%) and His309 (41%). Similar to itraconazole, ravuconazole consistently bound the heme iron, which was also stably bound to Cys471 throughout the simulation (Figure 12 and Supplementary Figure S9).

During simulation, montelukast, vilanterol, and lidoflazine interacted with at least one amino acid critical for maintaining the precise positioning of the heme group within the *Madurella mycetomatis* CYP51 active site. In the apo form of CYP51, Tyr122 and Tyr136 form hydrogen bonds with the carboxyl groups of the heme, playing a crucial role in stabilizing its position. Additionally, the sulfide group of Cys471 occupies the fifth coordination position of the heme iron atom, while Ile372 stabilizes the heme through hydrophobic interactions (Figure 2). Based on these observations, it is proposed that these drugs may disrupt CYP51’s catalytic activity by interfering with these key residues. Such interactions could obstruct substrate access or alter the electronic environment required for efficient heme electron transfer, potentially destabilizing the active site configuration [95]. Consequently, montelukast, vilanterol, and lidoflazine might inhibit or modulate *Madurella mycetomatis* CYP51 activity. These findings, combined with their systemic suitability, highlight their potential as promising candidates for repurposing in the treatment of eumycetoma.

### 2.5. Study Limitations and Future Perspectives

This study has several limitations and future directions to consider. Primarily, it relies on computational predictions through molecular docking and MM-GBSA calculations, which, while insightful, may not always reflect outcomes in complex biological systems. The homology model of *Madurella mycetomatis* CYP51, although validated, is based on computational modeling, as no experimentally resolved structure of this target is currently available. This limitation could introduce subtle inaccuracies affecting binding predictions. Furthermore, without experimental validation, the computationally identified drugs require in vitro and in vivo testing to confirm their efficacy and safety in treating eumycetoma. Moreover, obtaining an experimentally resolved structure of *Madurella mycetomatis* CYP51 would refine future in silico studies. Expansion to broader compound libraries and evaluating synergistic effects with other antifungal agents could further enhance therapeutic potential, while a focus on pharmacological and safety profiling will be essential, especially for off-target risks. Additionally, exploring other potential targets within *Madurella mycetomatis* could provide complementary or alternative therapeutic avenues to improve treatment outcomes for eumycetoma.

## 3. Materials and Methods

The homology model of *Madurella mycetomatis* CYP51 was built using the YASARA (Yet Another Scientific Artificial Reality Application) program [75]. All other computational studies were carried out in the Maestro interface within the Schrödinger suite (Schrödinger, LLC., New York, NY, USA, 2023). This included protein and ligand preparation, virtual screening, molecular docking, MM-GBSA calculations, and molecular dynamics (MD) simulations.

### 3.1. Model Building and Validation

The FASTA format file for the *Madurella mycetomatis* CYP51 amino acid sequence was obtained from the NCBI protein database (www.ncbi.nlm.nih.gov, accessed on 1 March 2023). The sequence was then used in a BLASTp search against proteins in the Protein Data Bank database (www.rcsb.org, accessed on 1 March 2023) to identify a template with the highest sequence identity and an experimentally determined structure. The molecular modeling program YASARA was used to build a homology model of the CYP51 enzyme [75]. The three-dimensional parameters of the constructed CYP51 homology model were validated by generating a Ramachandran plot of (φ) and (ψ) dihedral angles from the MolProbity server (http://molprobity.biochem.duke.edu, accessed on 2 March 2023) and checking the stereochemical accuracy [96]. The model folding reliability was assessed using the Verify3D tool (https://www.doe-mbi.ucla.edu, accessed on 5 March 2023), while the absolute quality was evaluated using the QMEAN z-score (https://swissmodel.expasy.org/qmean/, accessed on 9 March 2023) [97,98].

### 3.2. Computational Screening of Madurella mycetomatis CYP51 Inhibitors

A dataset of 2619 FDA-approved drugs was obtained from the DrugBank database (https://go.drugbank.com/, accessed on 25 March 2023) [76]. These drugs were prepared using the LigPrep tool (Schrödinger Release 2023-2: LigPrep, Schrödinger, LLC., New York, NY, USA, 2023), which generated and minimized 3D conformations, covering all possible tautomers and ionization states at pH 7.0 ± 2.0, using the OPLS4 force field. Preparation of the homology model of *Madurella mycetomatis* CYP51 employed Schrödinger’s Protein Preparation Wizard [78], involving hydrogen addition, bond order assignment, zero-order bonds to metals, disulfide bond formation, removal of water molecules beyond 5 Å from hetero groups, and capping of termini. Hydrogen bonding networks and water orientations were optimized, followed by structure refinement via minimization with the OPLS4 force field, with nonhydrogen atoms restrained to an RMSD of 0.3 Å. The heme was retained within the active site throughout this preparation [99]. The SiteMap tool (Schrödinger Release 2023-2: SiteMap, Schrödinger, LLC, New York, NY, USA, 2023) [100] was utilized to identify the binding pocket of the *Madurella mycetomatis* CYP51 homology model. The binding site with the highest Dscore (1.307) was selected for receptor grid generation and subsequent docking studies using the Glide program [79]. A receptor grid (10 Å × 10 Å × 10 Å) was created around the predicted binding site, adjusted to encompass the entire active site, using the Receptor Grid Generation tool in the Maestro suite with all other parameters set to their default values.

Virtual screening was conducted using the Glide program [79] with a flexible ligand docking approach on the prepared drug library, following the Virtual Screening Workflow (VSW) protocol [81]. Docking was performed in three stages, High-Throughput Virtual Screening (HTVS), Standard Precision (SP), and Extra Precision (XP), using default parameters. Drugs that successfully reached the XP stage were then rescored for binding energy using the Prime/MM-GBSA method [80]. The top three drugs, along with the reference drugs itraconazole and ravuconazole, were selected for further MD simulations using the Desmond simulation package of Schrödinger [101].

The MD simulations were performed on the apoprotein of the homology model of *Madurella mycetomatis* CYP51 and its complexes with montelukast, vilanterol, lidoflazine, ravuconazole, and itraconazole. The SPC solvation model was applied, and each system was placed in an orthorhombic water box with dimensions of 10 Å × 10 Å × 10 Å to ensure full solvation of each complex. Counter ions (Na^+^ or Cl^−^) were introduced to balance net charges, and 0.15 M NaCl was added to neutralize the systems. The systems were then minimized and pre-equilibrated using Desmond’s default relaxation protocol. The temperature was held at 300 K and the pressure was held at 1 bar, under the isothermal–isobaric (NPT) ensemble. A 100 ns simulation was conducted, capturing 1000 frames at 100 ps intervals.

## 4. Conclusions

In conclusion, this study utilized homology modeling and computational screening to identify FDA-approved drugs as promising candidates for repurposing against eumycetoma, with itraconazole serving as the reference drug. Montelukast, vilanterol, and lidoflazine were identified as potential candidates. These drugs demonstrated favorable binding affinities, robust interactions with key residues of the *Madurella mycetomatis* CYP51 homology model, and stability in MD simulations, suggesting their potential to effectively inhibit the target enzyme. Future studies should prioritize validating their efficacy and safety for treating eumycetoma through in vitro and in vivo investigations.

## Figures and Tables

**Table 1:** Docking scores, MM-GBSA energies, and clinical uses of the top-ranked FDA-approved drugs, along with itraconazole and ravuconazole, from the virtual screening workflow against the homology model of *Madurella mycetomatis* CYP51.

Drug	Clinical Use	Docking Score (kcal/mol)	MM-GBSA dG Bind (kcal/mol)
Bemotrizinol	Sunscreen	−12.536	−82.13
Vilanterol	Chronic obstructive pulmonary disease	−13.055	−66.69
Lidoflazine	Angina pectoris	−11.119	−62.76
Imatinib	Cancer	−11.079	−49.90
Montelukast	Asthma	−13.813	−48.82
Latanoprostene bunod	Glaucoma	−8.879	−48.15
Itraconazole (ionized)	Euycetoma	−5.115	−47.19
Carvedilol	Heart failure	−10.962	−44.39
Empagliflozin	Type 2 diabetes	−12.527	−43.85
Tazemetostat	Cancer	−10.141	−43.80
Polydatin	Anti-inflammatory, immunoregulatory, anti-oxidative, and anti-tumor activities	−13.581	−41.45
Axitinib	Cancer	−12.355	−40.51
Darifenacin	Urinary incontinence	−11.861	−37.62
Eliglustat	Type 1 Gaucher disease	−10.963	−37.08
Tezacaftor	Cystic fibrosis	−12.351	−36.16
Itraconazole (unionized)	Euycetoma	−7.049	−35.18
Ertugliflozin	Type 2 diabetes	−11.668	−33.88
Diosmin	Capillary fragility or venous insufficiency	−13.919	−32.07
Ravuconazole	Chagas disease	−5.625	−31.85
Dapagliflozin	Type 2 diabetes	−12.846	−20.24
Hesperidin	Blood vessel disorders	−13.593	−17.36
Etrasimod	Inflammatory bowel disease	−11.294	−8.12
Bortezomib D-mannitol	Multiple myeloma and mantle cell lymphoma	−10.874	22.90

**Table 2:** A detailed analysis of the PL-RMSD, P-RMSF, and protein–ligand contacts values, including minimum, maximum, and average for the complexes of itraconazole, ravuconazole, vilanterol, montelukast and lidoflazine with the homology model of *Madurella mycetomatis* CYP51. This also includes comparative data for the apoprotein.

CYP51 Complex	Apo	Itraconazole	Ravuconazole	Vilanterol	Montelukast	Lidoflazine
PL-RMSD (Å)						
average	3.1	3.8	3.3	2.8	3.0	3.2
maximum	4.2	4.5	4.0	3.5	3.7	4.2
minimum	1.5	1.3	1.4	1.3	1.2	1.2
P-RMSF (Å)						
average	1.2	1.2	1.1	1.1	1.1	1.3
maximum	9.7	6.4	8.4	8.6	8.8	10.2
minimum	0.5	0.5	0.4	0.4	0.4	0.4
H-bond contacts						
average	-	0.5	0.0	1.7	1.4	0.0
maximum	-	3.0	1.0	2.0	4.0	1.0
minimum	-	0.0	0.0	0.0	0.0	0.0
Hydrophobic contacts						
average	-	3.0	2.4	1.7	3.2	2.8
maximum	-	8.0	6.0	7.0	9.0	7.0
minimum	-	0.0	0.0	0.0	0.0	0.0

## Data Availability

The original contributions presented in the study are included in the article/Supplementary Material, and further inquiries can be directed to the corresponding authors.

## Supplementary Materials

The following supporting information can be downloaded at: https://www.mdpi.com/article/10.3390/ijms26010315/s1.

## References

[1] Zijlstra E.E., van de Sande W.W.J., Welsh O., Mahgoub E.S., Goodfellow M., Fahal A.H. (2016). Mycetoma: A unique neglected tropical disease. Lancet Infect. Dis..

[2] Elkheir L.Y.M., Haroun R., Mohamed M.A., Fahal A.H. (2020). *Madurella mycetomatis* causing eumycetoma medical treatment: The challenges and prospects. PLoS Negl. Trop. Dis..

[3] Relhan V., Mahajan K., Agarwal P., Garg V.K. (2017). Mycetoma: An Update. Indian J. Dermatol..

[4] Fahal A., Mahgoub E.L.S., Hassan A.M.E.L., Abdel-Rahman M.E. (2015). Mycetoma in the Sudan: An Update from the Mycetoma Research Centre, University of Khartoum, Sudan. PLoS Negl. Trop. Dis..

[5] Elkheir L.Y.M., Delaye P.-O., Penichon M., Eadie K., Mohamed M.A., Besson P., Chesnay A., Desoubeaux G., Roger S., van de Sande W.W.J. (2024). Emerging therapeutics: The imidazo[1,2-b]pyridazine scaffold as a novel drug candidate for eumycetoma, a neglected tropical disease. Eur. J. Med. Chem..

[6] Suleiman S.H., Wadaella el S., Fahal A.H. (2016). The Surgical Treatment of Mycetoma. PLoS Negl. Trop. Dis..

[7] Agarwal P., Jagati A., Rathod S.P., Kalra K., Patel S., Chaudhari M. (2021). Clinical Features of Mycetoma and the Appropriate Treatment Options. Res. Rep. Trop. Med..

[8] Efared B., Tahiri L., Boubacar M.S., Atsam-Ebang G., Hammas N., Hinde E.F., Chbani L. (2017). Mycetoma in a non-endemic area: A diagnostic challenge. BMC Clin. Pathol..

[9] Hao X., Cognetti M., Burch-Smith R., Mejia E.O., Mirkin G. (2022). Mycetoma: Development of Diagnosis and Treatment. J. Fungi.

[10] Clark J.E., Kim H.Y., van de Sande W.W.J., McMullan B., Verweij P., Alastruey-Izquierdo A., Chakrabarti A., Harrison T.S., Bongomin F., Hay R.J. (2024). Eumycetoma causative agents: A systematic review to inform the World Health Organization priority list of fungal pathogens. Med. Mycol..

[11] Grover S., Roy P., Singh G. (2001). MADURA FOOT. Med. J. Armed Forces India.

[12] Alam K., Maheshwari V., Bhargava S., Jain A., Fatima U., Haq E.U. (2009). Histological diagnosis of madura foot (mycetoma): A must for definitive treatment. J. Glob. Infect. Dis..

[13] Chandler D.J., Bonifaz A., van de Sande W.W.J. (2023). An update on the development of novel antifungal agents for eumycetoma. Front. Pharmacol..

[14] Hashizume H., Taga S., Sakata M.K., Hussein M., Siddig E.E., Minamoto T., Fahal A.H., Kaneko S. (2023). Environmental detection of eumycetoma pathogens using multiplex real-time PCR for soil DNA in Sennar State, Sudan. Trop. Med. Health.

[15] Welsh O., Al-Abdely H.M., Salinas-Carmona M.C., Fahal A.H. (2014). Mycetoma medical therapy. PLoS Negl. Trop. Dis..

[16] Salim A.O., Mwita C.C., Gwer S. (2018). Treatment of Madura foot: A systematic review. JBI Database Syst. Rev. Implement. Rep..

[17] Siddig E.E., Ahmed A., Ali Y., Bakhiet S.M., Mohamed N.S., Ahmed E.S., Fahal A.H. (2021). Eumycetoma Medical Treatment: Past, Current Practice, Latest Advances and Perspectives. Microbiol. Res..

[18] Venugopal P.V., Venugopal T.V. (1993). Treatment of eumycetoma with ketoconazole. Australas. J. Dermatol..

[19] Paugam A., Tourte-Schaefer C., Keïta A., Chemla N., Chevrot A. (1997). Clinical cure of fungal madura foot with oral itraconazole. Cutis.

[20] Lacroix C., de Kerviler E., Morel P., Derouin F., Feuilhade de Chavin M. (2005). *Madurella mycetomatis* mycetoma treated successfully with oral voriconazole. Br. J. Dermatol..

[21] Fahal A.H., Rahman I.A., El-Hassan A.M., Rahman M.E., Zijlstra E.E. (2011). The safety and efficacy of itraconazole for the treatment of patients with eumycetoma due to *Madurella mycetomatis*. Trans. R. Soc. Trop. Med. Hyg..

[22] Loulergue P., Hot A., Dannaoui E., Dallot A., Poirée S., Dupont B., Lortholary O. (2006). Successful treatment of black-grain mycetoma with voriconazole. Am. J. Trop. Med. Hyg..

[23] Negroni R., Tobón A., Bustamante B., Shikanai-Yasuda M.A., Patino H., Restrepo A. (2005). Posaconazole treatment of refractory eumycetoma and chromoblastomycosis. Rev. Inst. Med. Trop. Sao Paulo.

[24] Castro L.G., Piquero-Casals J. (2008). Clinical and mycologic findings and therapeutic outcome of 27 mycetoma patients from São Paulo, Brazil. Int. J. Dermatol..

[25] N’Diaye B., Dieng M.T., Perez A., Stockmeyer M., Bakshi R. (2006). Clinical efficacy and safety of oral terbinafine in fungal mycetoma. Int. J. Dermatol..

[26] Emmanuel P., Dumre S.P., John S., Karbwang J., Hirayama K. (2018). Mycetoma: A clinical dilemma in resource limited settings. Ann. Clin. Microbiol. Antimicrob..

[27] Mattioni S., Develoux M., Brun S., Martin A., Jaureguy F., Naggara N., Bouchaud O. (2013). Management of mycetomas in France. Med. Mal. Infect..

[28] Siddig E.E., Ahmed A. (2024). The urgent need for developing and implementing a multisectoral One Health strategy for the surveillance, prevention, and control of Eumycetoma. IJID One Health.

[29] Mahgoub E.S., Gumaa S.A. (1984). Ketoconazole in the treatment of eumycetoma due to *Madurella mycetomii*. Trans. R. Soc. Trop. Med. Hyg..

[30] Gupta A.K., Lyons D.C. (2015). The Rise and Fall of Oral Ketoconazole. J. Cutan. Med. Surg..

[31] van de Sande W.W.J., de Kat J., Coppens J., Ahmed A.O.A., Fahal A., Verbrugh H., van Belkum A. (2007). Melanin biosynthesis in *Madurella mycetomatis* and its effect on susceptibility to itraconazole and ketoconazole. Microbes Infect..

[32] Geneugelijk K., Kloezen W., Fahal A.H., van de Sande W.W.J. (2014). Active Matrix Metalloprotease-9 Is Associated with the Collagen Capsule Surrounding the *Madurella mycetomatis* Grain in Mycetoma. PLoS Negl. Trop. Dis..

[33] van de Sande W.W., Maghoub el S., Fahal A.H., Goodfellow M., Welsh O., Zijlstra E. (2014). The mycetoma knowledge gap: Identification of research priorities. PLoS Negl. Trop. Dis..

[34] Gismalla M.D.A., Bakhiet M.Y., Alshareef A.M., Saadeldien M.S.M., Ahmed G.M.A., Adam A.M.I., Abuelnour A.E.K. (2024). Reconstructive surgery for mycetoma: Preliminary algorithm and a systematic review. JPRAS Open.

[35] Scolding P., Fahal A., Yotsu R.R. (2018). Drug therapy for Mycetoma. Cochrane Database Syst. Rev..

[36] Fahal A.H., Ahmed E.S., Bakhiet S.M., Bakhiet O.E., Fahal L.A., Mohamed A.A., Mohamedelamin E.S.W., Bahar M.E.N., Attalla H.Y., Siddig E.E. (2024). Two dose levels of once-weekly fosravuconazole versus daily itraconazole in combination with surgery in patients with eumycetoma in Sudan: A randomised, double-blind, phase 2, proof-of-concept superiority trial. Lancet Infect. Dis..

[37] Fahal A.H., Shaheen S., Jones D.H.A. (2014). The orthopaedic aspects of mycetoma. Bone Jt. J..

[38] Fahal A.H., Ahmed K.O., Saeed A.A., Elkhawad A.O., Bakhiet S.M. (2022). Why the mycetoma patients are still neglected. PLoS Negl. Trop. Dis..

[39] Yamey G., Torreele E. (2002). The world’s most neglected diseases. BMJ.

[40] Weng H.B., Chen H.X., Wang M.W. (2018). Innovation in neglected tropical disease drug discovery and development. Infect. Dis. Poverty.

[41] Smit S., Derks M.F., Bervoets S., Fahal A., van Leeuwen W., van Belkum A., van de Sande W.W. (2016). Genome Sequence of *Madurella mycetomatis* mm55, Isolated from a Human Mycetoma Case in Sudan. Genome Announc..

[42] Chatelain E., Ioset J.R. (2011). Drug discovery and development for neglected diseases: The DNDi model. Drug Des. Dev. Ther..

[43] Lim W., Eadie K., Konings M., van de Sande W. (2020). MycetOS—An open research model discover new drugs to treat one of the most neglected disease—Mycetoma. Int. J. Infect. Dis..

[44] Murray A.J., Cox L.R., Adcock H.V., Roberts R.A. (2024). Academic drug discovery: Challenges and opportunities. Drug Discov. Today.

[45] Calderone R., Sun N., Gay-Andrieu F., Groutas W., Weerawarna P., Prasad S., Alex D., Li D. (2014). Antifungal drug discovery: The process and outcomes. Future Microbiol..

[46] Elbadawi M.A., Awadalla M.K., Hamid M.M., Mohamed M.A., Awad T.A. (2015). Valproic acid as a potential inhibitor of *Plasmodium falciparum* histone deacetylase 1 (PfHDAC1): An in silico approach. Int. J. Mol. Sci..

[47] Mohamed M.A., Alanazi A.F., Alanazi W.A., Elsaman T., Mohamed M.S., Eltayib E.M. (2024). Repurposing of eluxadoline as a SARS-CoV-2 main protease inhibitor: E-Pharmacophore based virtual screening, molecular docking, MM-GBSA calculations, and molecular dynamics simulations studies. J. Appl. Pharm. Sci..

[48] Mohamed A.A.E., Mohamed K.A.A., Marwa S.S.O., Magdi A.M., Mahmoud M.E.M., Muzamil M.A.H., Malik S.M., Mohammed A.G. (2016). Evaluation of antileishmanial activity of valproic acid against *Leishmania donovani*: An integrated in silico and in vitro study. World J. Pharm. Sci..

[49] Elsaman T., Ahmad I., Eltayib E.M., Suliman Mohamed M., Yusuf O., Saeed M., Patel H., Mohamed M.A. (2024). Flavonostilbenes natural hybrids from *Rhamnoneuron balansae* as potential antitumors targeting ALDH1A1: Molecular docking, ADMET, MM-GBSA calculations and molecular dynamics studies. J. Biomol. Struct. Dyn..

[50] Medina-Franco J.L. (2021). Grand Challenges of Computer-Aided Drug Design: The Road Ahead. Drug Discov..

[51] Gurung A.B., Ali M.A., Lee J., Farah M.A., Al-Anazi K.M. (2021). An Updated Review of Computer-Aided Drug Design and Its Application to COVID-19. BioMed Res. Int..

[52] Chang Y., Hawkins B.A., Du J.J., Groundwater P.W., Hibbs D.E., Lai F. (2022). A Guide to In Silico Drug Design. Pharmaceutics.

[53] Kuntz I.D., Blaney J.M., Oatley S.J., Langridge R., Ferrin T.E. (1982). A geometric approach to macromolecule-ligand interactions. J. Mol. Biol..

[54] Stanzione F., Giangreco I., Cole J.C. (2021). Use of molecular docking computational tools in drug discovery. Prog. Med. Chem..

[55] Meng X.Y., Zhang H.X., Mezei M., Cui M. (2011). Molecular docking: A powerful approach for structure-based drug discovery. Curr. Comput.-Aided Drug Des..

[56] Zhang B., Li H., Yu K., Jin Z. (2022). Molecular docking-based computational platform for high-throughput virtual screening. CCF Trans. High Perform. Comput..

[57] Alvarez J.C. (2004). High-throughput docking as a source of novel drug leads. Curr. Opin. Chem. Biol..

[58] Mohamed M.A., Elsaman T., Mohamed M.S., Eltayib E.M. (2024). Computational investigations of flavonoids as ALDH isoform inhibitors for treatment of cancer. SAR QSAR Environ. Res..

[59] Mohanty M., Mohanty P.S. (2023). Molecular docking in organic, inorganic, and hybrid systems: A tutorial review. Monatshefte Chem.-Chem. Mon..

[60] Agu P.C., Afiukwa C.A., Orji O.U., Ezeh E.M., Ofoke I.H., Ogbu C.O., Ugwuja E.I., Aja P.M. (2023). Molecular docking as a tool for the discovery of molecular targets of nutraceuticals in diseases management. Sci. Rep..

[61] Huang S.Y., Zou X. (2010). Advances and challenges in protein-ligand docking. Int. J. Mol. Sci..

[62] Smyth M.S., Martin J.H. (2000). x ray crystallography. Mol. Pathol..

[63] Muhammed M.T., Aki-Yalcin E. (2019). Homology modeling in drug discovery: Overview, current applications, and future perspectives. Chem. Biol. Drug Des..

[64] Awad T.A., Abd Algaffar S.O., Van de Sande W.W., Khalid S.A. (2018). Molecular Docking Based on Construction of 4-α-Sterol Demethylase (CYP51) as an Active Site of *Madurella mycetomatis* by Homology Modelling. https://www.morressier.com/o/event/5abccf42d462b8028d899a8f/article/5ac39997d462b8028d89a296.

[65] Can N.Ö., Acar Çevik U., Sağlık B.N., Levent S., Korkut B., Özkay Y., Kaplancıklı Z.A., Koparal A.S. (2017). Synthesis, Molecular Docking Studies, and Antifungal Activity Evaluation of New Benzimidazole-Triazoles as Potential Lanosterol 14α-Demethylase Inhibitors. J. Chem..

[66] Sagatova A.A. (2021). Strategies to Better Target Fungal Squalene Monooxygenase. J. Fungi.

[67] Rodrigues M.L. (2018). The Multifunctional Fungal Ergosterol. mBio.

[68] Teixeira M.M., Carvalho D.T., Sousa E., Pinto E. (2022). New Antifungal Agents with Azole Moieties. Pharmaceuticals.

[69] Hoobler E.K., Rai G., Warrilow A.G., Perry S.C., Smyrniotis C.J., Jadhav A., Simeonov A., Parker J.E., Kelly D.E., Maloney D.J. (2013). Discovery of a novel dual fungal CYP51/human 5-lipoxygenase inhibitor: Implications for anti-fungal therapy. PLoS ONE.

[70] Sheng C., Zhang W., Zhang M., Song Y., Ji H., Zhu J., Yao J., Yu J., Yang S., Zhou Y. (2004). Homology Modeling of Lanosterol 14α-Demethylase of Candida albicans and Aspergillus fumigatus and Insights into the Enzyme-Substrate Interactions. J. Biomol. Struct. Dyn..

[71] Warrilow A.G., Mullins J.G., Hull C.M., Parker J.E., Lamb D.C., Kelly D.E., Kelly S.L. (2012). S279 point mutations in Candida albicans Sterol 14-α demethylase (CYP51) reduce in vitro inhibition by fluconazole. Antimicrob. Agents Chemother..

[72] Güzel E., Acar Çevik U., Evren A.E., Bostancı H.E., Gül Ü.D., Kayış U., Özkay Y., Kaplancıklı Z.A. (2023). Synthesis of Benzimidazole-1,2,4-triazole Derivatives as Potential Antifungal Agents Targeting 14α-Demethylase. ACS Omega.

[73] Sagatova A.A., Keniya M.V., Wilson R.K., Sabherwal M., Tyndall J.D., Monk B.C. (2016). Triazole resistance mediated by mutations of a conserved active site tyrosine in fungal lanosterol 14α-demethylase. Sci. Rep..

[74] Jiang Y., Zhang J., Cao Y., Chai X., Zou Y., Wu Q., Zhang D., Jiang Y., Sun Q. (2011). Synthesis, in vitro evaluation and molecular docking studies of new triazole derivatives as antifungal agents. Bioorg. Med. Chem. Lett..

[75] Krieger E., Vriend G., Spronk C. YASARA–Yet Another Scientific Artificial Reality Application. YASARA org 2013. Volume 993, pp. 51–78. http://www.yasara.org/general.htm.

[76] Knox C., Wilson M., Klinger C.M., Franklin M., Oler E., Wilson A., Pon A., Cox J., Chin N.E.L., Strawbridge S.A. (2024). DrugBank 6.0: The DrugBank Knowledgebase for 2024. Nucleic Acids Res..

[77] Friggeri L., Hargrove T.Y., Wawrzak Z., Blobaum A.L., Rachakonda G., Lindsley C.W., Villalta F., Nes W.D., Botta M., Guengerich F.P. (2018). Sterol 14α-Demethylase Structure-Based Design of VNI ((R)-N-(1-(2,4-Dichlorophenyl)-2-(1 H-imidazol-1-yl)ethyl)-4-(5-phenyl-1,3,4-oxadiazol-2-yl)benzamide)) Derivatives To Target Fungal Infections: Synthesis, Biological Evaluation, and Crystallographic Analysis. J. Med. Chem..

[78] Madhavi Sastry G., Adzhigirey M., Day T., Annabhimoju R., Sherman W. (2013). Protein and ligand preparation: Parameters, protocols, and influence on virtual screening enrichments. J. Comput.-Aided Mol. Des..

[79] Yang Y., Yao K., Repasky M.P., Leswing K., Abel R., Shoichet B.K., Jerome S.V. (2021). Efficient Exploration of Chemical Space with Docking and Deep Learning. J. Chem. Theory Comput..

[80] Jacobson M.P., Pincus D.L., Rapp C.S., Day T.J.F., Honig B., Shaw D.E., Friesner R.A. (2004). A hierarchical approach to all-atom protein loop prediction. Proteins Struct. Funct. Bioinform..

[81] Pandya V., Rao P., Prajapati J., Rawal R.M., Goswami D. (2024). Pinpointing top inhibitors for GSK3β from pool of indirubin derivatives using rigorous computational workflow and their validation using molecular dynamics (MD) simulations. Sci. Rep..

[82] Mohamed M.A., Elsaman T., Elderdery A.Y., Alsrhani A., Ghanem H.B., Alruwaili M.M., Hamza S.M.A., Mekki S.E.I., Alotaibi H.A., Mills J. (2024). Unveiling the Anticancer Potential: Computational Exploration of Nitrogenated Derivatives of (+)-Pancratistatin as Topoisomerase I Inhibitors. Int. J. Mol. Sci..

[83] Benson H.A. (2000). Assessment and clinical implications of absorption of sunscreens across skin. Am. J. Clin. Dermatol..

[84] Siddiqui M., Rajkumar S.V. (2012). The high cost of cancer drugs and what we can do about it. Mayo Clin. Proc..

[85] Hoy S.M. (2018). Latanoprostene Bunod Ophthalmic Solution 0.024%: A Review in Open-Angle Glaucoma and Ocular Hypertension. Drugs.

[86] Alqahtani M.S., Kazi M., Alsenaidy M.A., Ahmad M.Z. (2021). Advances in Oral Drug Delivery. Front. Pharmacol..

[87] Dwan K., Milan S.J., Bax L., Walters N., Powell C. (2016). Vilanterol and fluticasone furoate for asthma. Cochrane Database Syst. Rev..

[88] Ridley J.M., Dooley P.C., Milnes J.T., Witchel H.J., Hancox J.C. (2004). Lidoflazine is a high affinity blocker of the HERG K^+^ channel. J. Mol. Cell. Cardiol..

[89] McCarthy M.W. (2023). Montelukast as a potential treatment for COVID-19. Expert Opin. Pharmacother..

[90] Rashid H.U., Ahmad N., Abdalla M., Khan K., Martines M.A.U., Shabana S. (2022). Molecular docking and dynamic simulations of Cefixime, Etoposide and Nebrodenside A against the pathogenic proteins of SARS-CoV-2. J. Mol. Struct..

[91] Stodola T.J., Chi Y.I., De Assuncao T.M., Leverence E.N., Tripathi S., Dsouza N.R., Mathison A.J., Volkman B.F., Smith B.C., Lomberk G. (2022). Computational modeling reveals key molecular properties and dynamic behavior of disruptor of telomeric silencing 1-like (DOT1L) and partnering complexes involved in leukemogenesis. Proteins.

[92] Ayaz P., Lyczek A., Paung Y., Mingione V.R., Iacob R.E., de Waal P.W., Engen J.R., Seeliger M.A., Shan Y., Shaw D.E. (2023). Structural mechanism of a drug-binding process involving a large conformational change of the protein target. Nat. Commun..

[93] Patil R., Das S., Stanley A., Yadav L., Sudhakar A., Varma A.K. (2010). Optimized hydrophobic interactions and hydrogen bonding at the target-ligand interface leads the pathways of drug-designing. PLoS ONE.

[94] Niu X., Lin L., Liu L., Yu Y., Wang H. (2022). Antifungal activity and molecular mechanisms of mulberrin derivatives against *Colletotrichum gloeosporioides* for mango storage. Int. J. Food Microbiol..

[95] Hargrove T.Y., Wawrzak Z., Liu J., Waterman M.R., Nes W.D., Lepesheva G.I. (2012). Structural complex of sterol 14α-demethylase (CYP51) with 14α-methylenecyclopropyl-Delta7-24, 25-dihydrolanosterol. J. Lipid Res..

[96] Williams C.J., Headd J.J., Moriarty N.W., Prisant M.G., Videau L.L., Deis L.N., Verma V., Keedy D.A., Hintze B.J., Chen V.B. (2018). MolProbity: More and better reference data for improved all-atom structure validation. Protein Sci..

[97] Lüthy R., Bowie J.U., Eisenberg D. (1992). Assessment of protein models with three-dimensional profiles. Nature.

[98] Benkert P., Künzli M., Schwede T. (2009). QMEAN server for protein model quality estimation. Nucleic Acids Res..

[99] Vemula D., Maddi D.R., Bhandari V. (2023). Homology modeling, virtual screening, molecular docking, and dynamics studies for discovering *Staphylococcus epidermidis* FtsZ inhibitors. Front. Mol. Biosci..

[100] Halgren T.A. (2009). Identifying and Characterizing Binding Sites and Assessing Druggability. J. Chem. Inf. Model..

[101] Bowers K.J., Chow D.E., Xu H., Dror R.O., Eastwood M.P., Gregersen B.A., Klepeis J.L., Kolossvary I., Moraes M.A., Sacerdoti F.D. Scalable Algorithms for Molecular Dynamics Simulations on Commodity Clusters. Proceedings of the SC ’06: Proceedings of the 2006 ACM/IEEE Conference on Supercomputing.

