# Supplementary material for: Repurposing FDA-Approved Drugs for Eumycetoma Treatment: Homology Modeling and Computational Screening of CYP51 Inhibitors
Source: Int J Mol Sci. 2025 Jan 1;26(1):315. doi: 10.3390/ijms26010315 (PMC11720416; doi:10.3390/ijms26010315)
Supplement: Supplementary file 1 [file ijms-26-00315-s001.zip › ijms-3382807-supplementary.pdf]

Repurposing FDA-Approved Drugs for Eumycetoma Treatment: Homology Modeling and Computational Screening of CYP51 Inhibitors

Magdi Awadalla Mohamed,[1†*] Mohamed Khalid Alhaj Awadalla,[2†] Malik Suliman Mohamed,[3] Tilal Elsaman,[1*] Eyman Mohamed Eltayib[3]

[1]*Department of Pharmaceutical Chemistry, College of Pharmacy, Jouf University, Sakaka 72388, Saudi Arabia*

[2]*Pharmacy Program, Wad Medani College of Medical Sciences and Technology, Wad Medani 21111, Gezira, Sudan*

[3]*Department of Pharmaceutics, College of Pharmacy, Jouf University, Sakaka 72388, Saudi Arabia*

[†]*These authors contributed equally to this work*

[*]*To whom correspondence should be addressed: MAM (maelhussein@ju.edu.sa) and TE (telbashir@ju.edu.sa)*

```
Query: Eburicol 14-alpha-demethylase [Madurella mycetomatis] Query ID: KXX80456.1 Length: 529


>14-alpha sterol demethylase Cyp51B [Aspergillus fumigatus Af293]
Sequence ID: EAL87096.1 Length: 524
Range 1: 1 to 519

Score:702 bits(1811), Expect:0.0,
Method:Compositional matrix adjust.,
Identities:328/528(62%), Positives:420/528(79%), Gaps:10/528(1%)

Query  1    MGLVQDIASPLAEGFSRFGLASQIGIAFGVFLFVAVLLNVLQQVLFRNPNEPPLVFHWFP  60
            MGL+  I   + +  S       +GI     L V+V++NVLQQ+LF+NP+EPP+VFHWFP
Sbjct  1    MGLIAFILDGICKHCSTQSTWVLVGIGLLSILAVSVIINVLQQLLFKNPHEPPVVFHWFP  60

Query  61   LIGSTITYGMDPPRFFKENRAKYGECFTFVLLGKKTTVYLGTQGNDFILNGKIRDVCAEE  120
             IGSTI+YG+DP +FF + RAKYG+ FTF+LLGKKTTVYLGT+GNDFILNGK+RDVCAEE
Sbjct  61   FIGSTISYGIDPYKFFFDCRAKYGDIFTFILLGKKTTVYLGTKGNDFILNGKLRDVCAEE  120

Query  121  IYTVLTTPVFGKDVVYDCPNSKLMEQKKFMKVALTTDAFRSYVPIISDEVTSYFKRTSDF  180
            +Y+ LTTPVFG+ VVYDCPN+KLMEQKKF+K  LT+DA RSYVP+I+DEV S+ K +  F
Sbjct  121  VYSPLTTPVFGRHVVYDCPNAKLMEQKKFVKYGLTSDALRSYVPLITDEVESFVKNSPAF  180

Query  181  KGQSGIVNICPKMAQITIFTASHALQGKEIRSKFDESLADLYHDLDMGFSPINFKLHWAP  240
            +G  G+ ++C  +A+ITI+TAS +LQGKE+RSKFD + A+LYH+LDMGF+PINF L WAP
Sbjct  181  QGHKGVFDVCKTIAEITIYTASRSLQGKEVRSKFDSTFAELYHNLDMGFAPINFMLPWAP  240

Query  241  LPWNQRRDHAQRTIAKIYMDTIKSRRARG-ETDAKDIMWHLMNSEYKNGVKVPDHEIAHM  299
            LP N++RD AQR + + YM+ IK+RR  G + D++D++W+LM+  YKNG  VPD EIAHM
Sbjct  241  LPHNRKRDAAQRKLTETYMEIIKARRQAGSKKDSEDMVWNLMSCVYKNGTPVPDEEIAHM  300

Query  300  MIALLMAGQHSSSSTSSWIMLRLASRPDIMEELYQEQVKNLGADLPPLKYEDLAKLPLNQ  359
            MIALLMAGQHSSSST+SWI+LRLA+RPDIMEELYQEQ++ LG+DLPPL Y++L KL L+
Sbjct  301  MIALLMAGQHSSSSTASWIVLRLATRPDIMEELYQEQIRVLGSDLPPLTYDNLQKLDLHA  360

Query  360  AIVKETLRLHAPIHSIMRAVKQPMPIPGTKYVIPTNHVLLAAPGVSASDPQYFPEPDLWE  419
             ++KETLRLHAPIHSI+RAVK PM + GT YVIPT+H +L++PGV+A   ++FP P  W
Sbjct  361  KVIKETLRLHAPIHSIIRAVKNPMAVDGTSYVIPTSHNVLSSPGVTARSEEHFPNPLEWN  420

Query  420  PHRWEKESPLAPSIVRNEAAEEDEEKVDYGYGLVSKGAGSPYLPFGAGRHRCIGEQFANV  479
            PHRW++            A+ ED+EKVDYGYGLVSKG  SPYLPFGAGRHRCIGEQFA +
Sbjct  421  PHRWDENI---------AASAEDDEKVDYGYGLVSKGTNSPYLPFGAGRHRCIGEQFAYL  471

Query  480  QLQTIVAMTVRLFKFRNVDSSNKVIGTDYASLFSRPLEPANIYWERRD  527
            QL TI A+ VRLF+FRN+   + +  TDY+SLFS+PL  + + +E+R+
Sbjct  472  QLGTITAVLVRLFRFRNLPGVDGIPDTDYSSLFSKPLGRSFVEFEKRE  519
```

Supplementary Figure S1: Pairwise sequence alignment of *Madurella mycetomatis* CYP51 (query) with *Aspergillus fumigatus* CYP51B (subject).

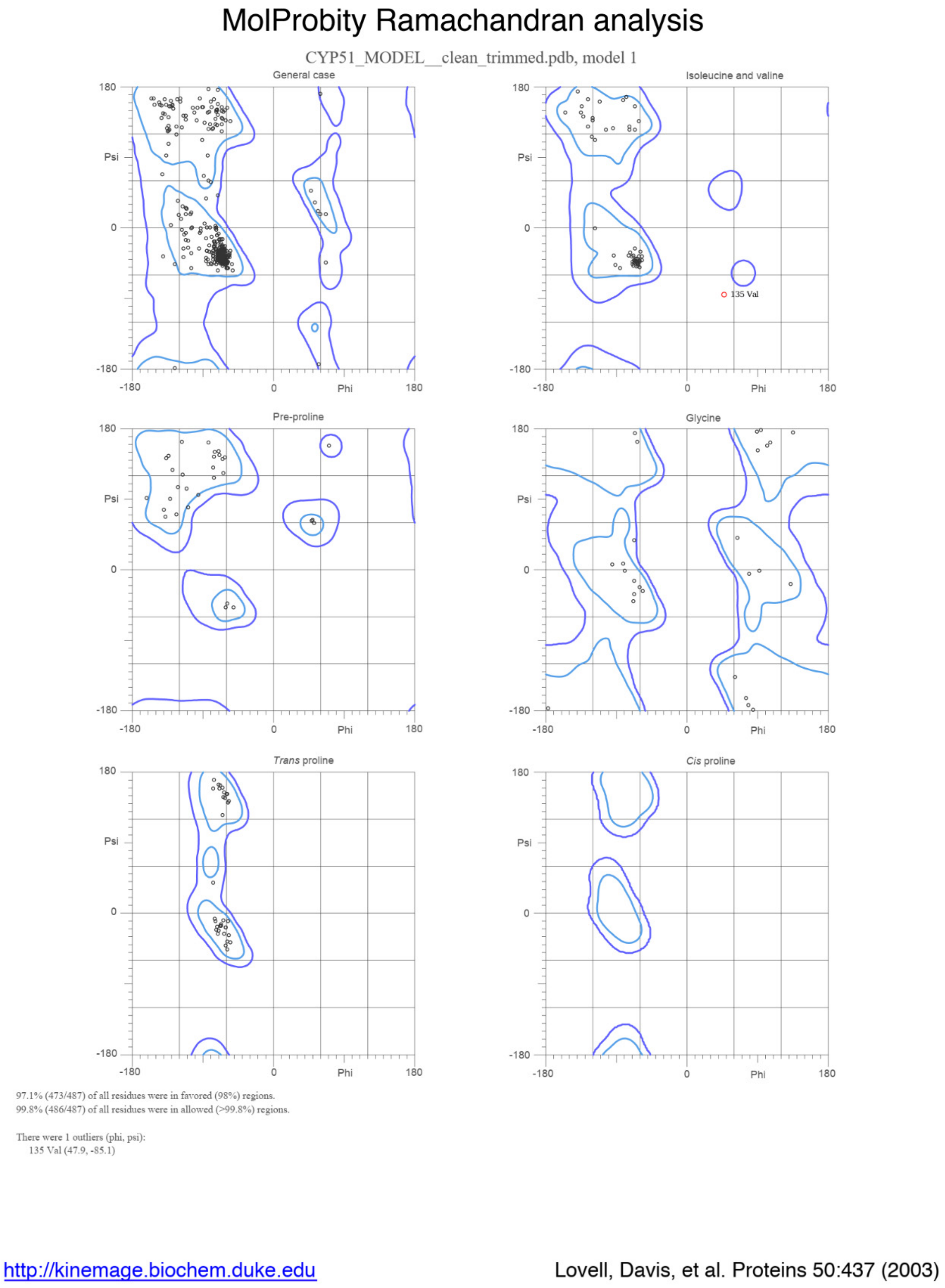

Supplementary Figure S2a: Ramachandran plot of the φ and ψ angles of the *Madurella mycetomatis* CYP51 homology model.

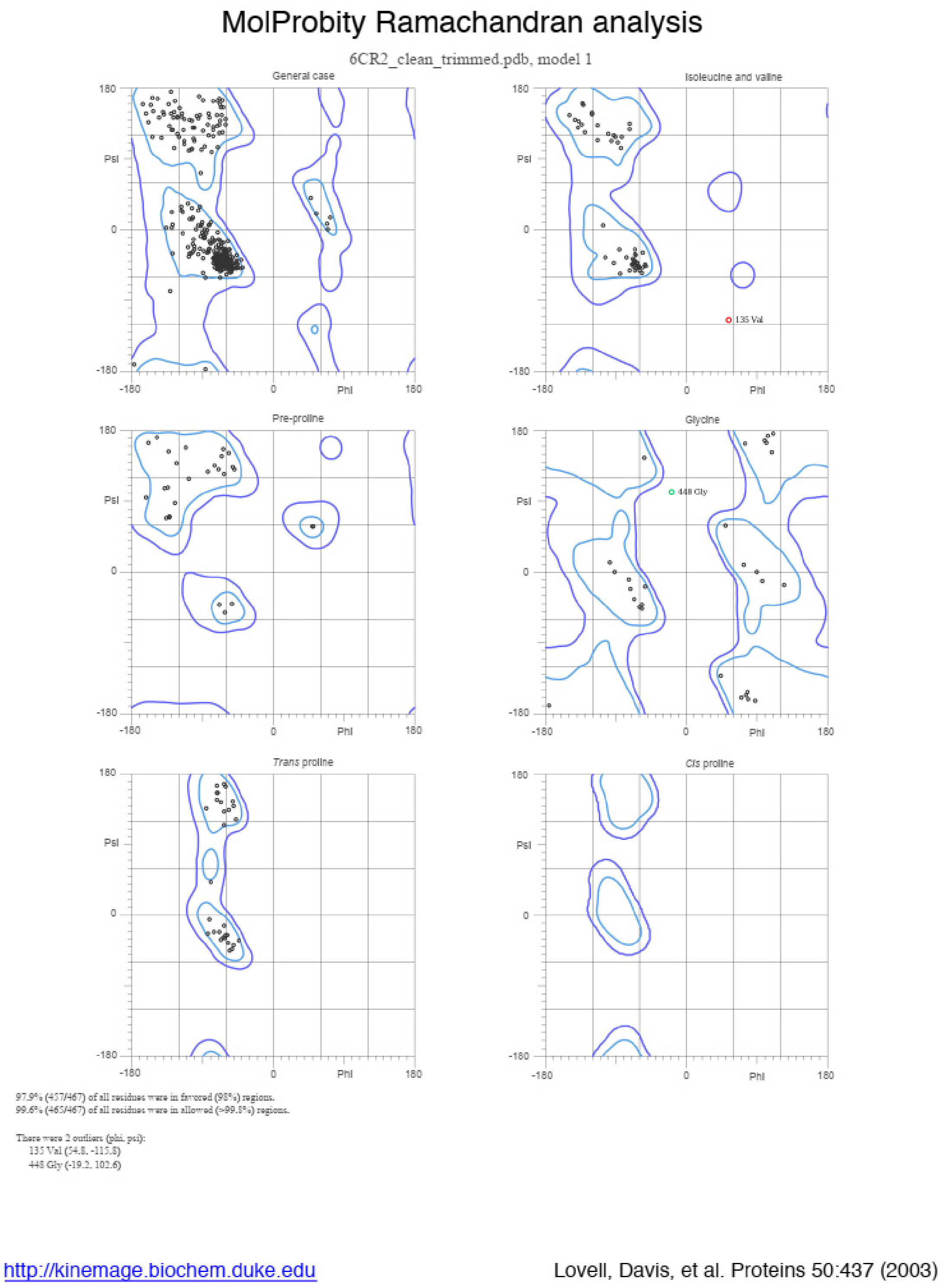

Supplementary Figure S2b: Ramachandran plot of the φ and ψ angles of *Aspergillus fumigatus* CYP51B (PDB ID: 6CR2).

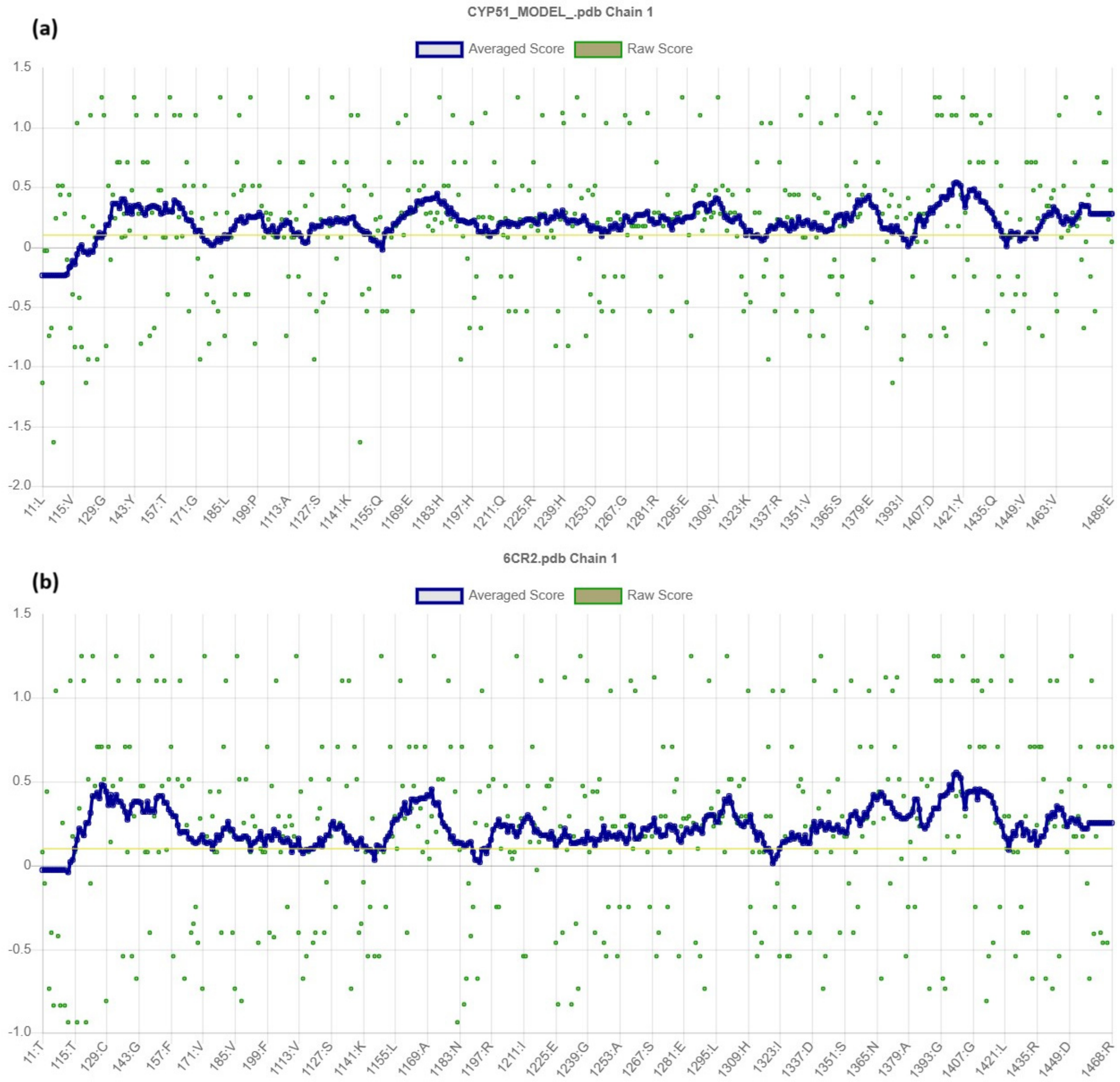

Supplementary Figure S3: Verify3D-1D plot for (a) *Madurella mycetomatis* CYP51 homology model and (b) *Aspergillus fumigatus* CYP51B (PDB ID: 6CR2).

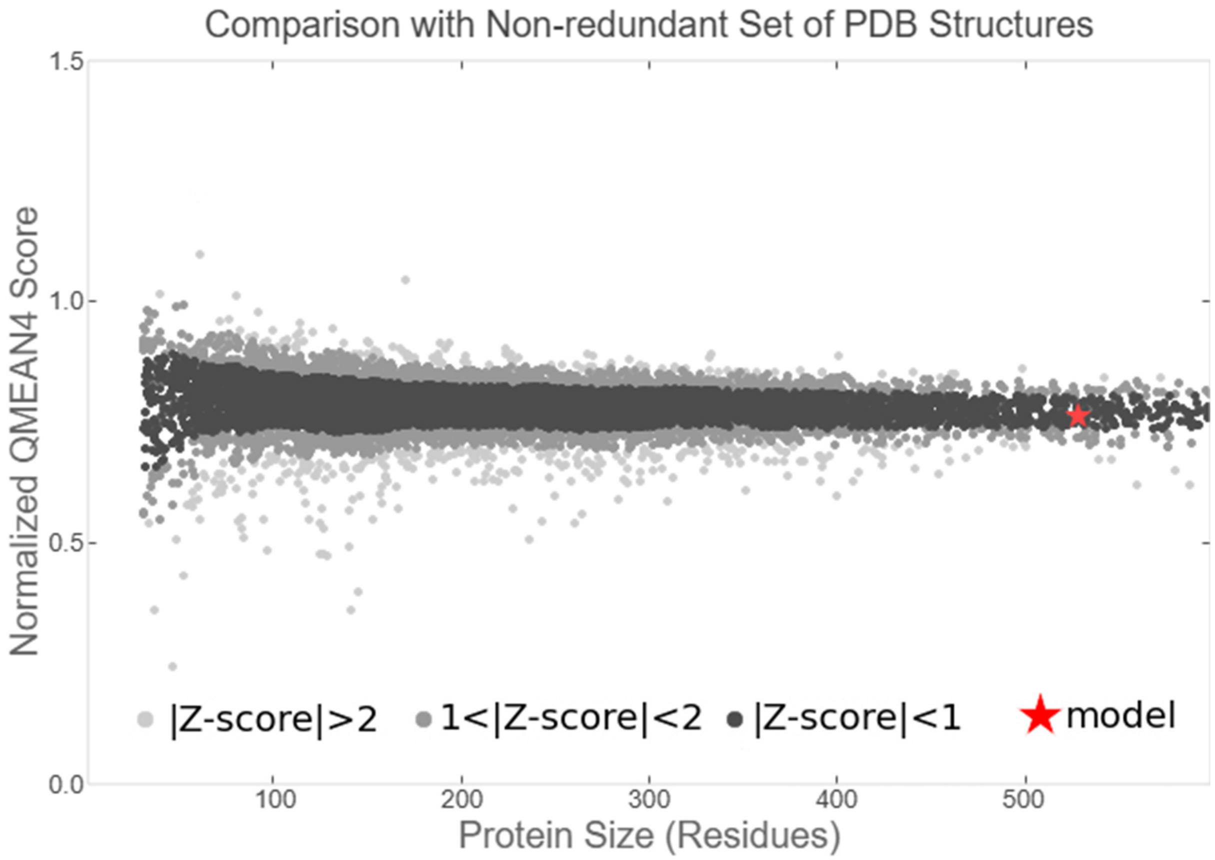

Supplementary Figure S4: The Qualitative Model Energy ANalysis (QMEAN) for the *Madurella mycetomatis* CYP51 homology model.

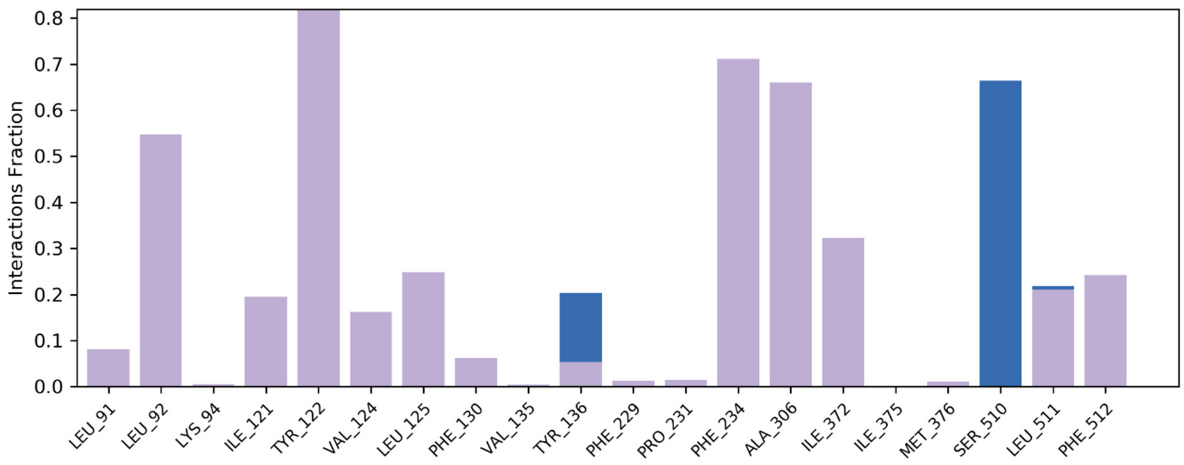

Supplementary Figure S5: Interactions of the *Madurella mycetomatis* CYP51 homology model residues with lidoflazine during the entire MD simulations run. Two interactions were observed: water bridges (blue) and hydrophobic interactions (grey).

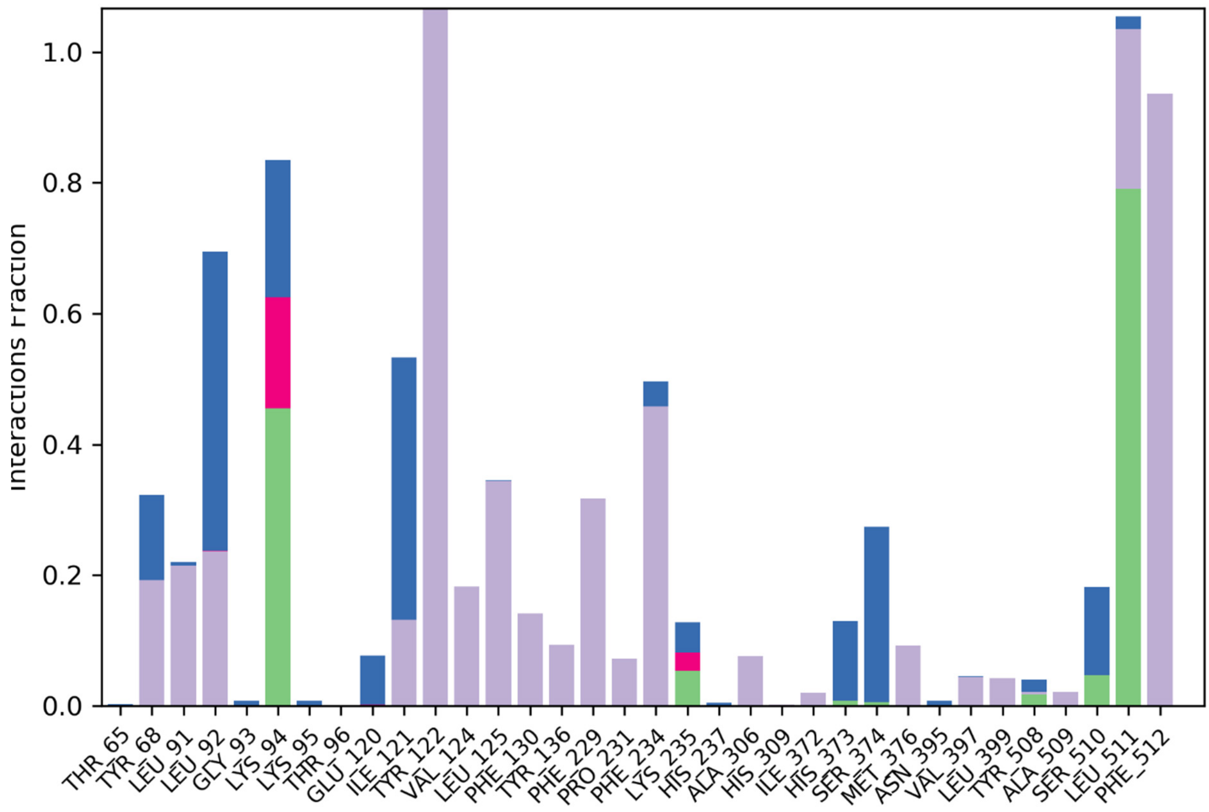

Supplementary Figure S6: Interactions of the *Madurella mycetomatis* CYP51 homology model residues with montelukast during the entire MD simulations run. Different interactions were observed: hydrogen bonds (green); water bridges (blue), hydrophobic interactions (grey); and ionic interactions (red).

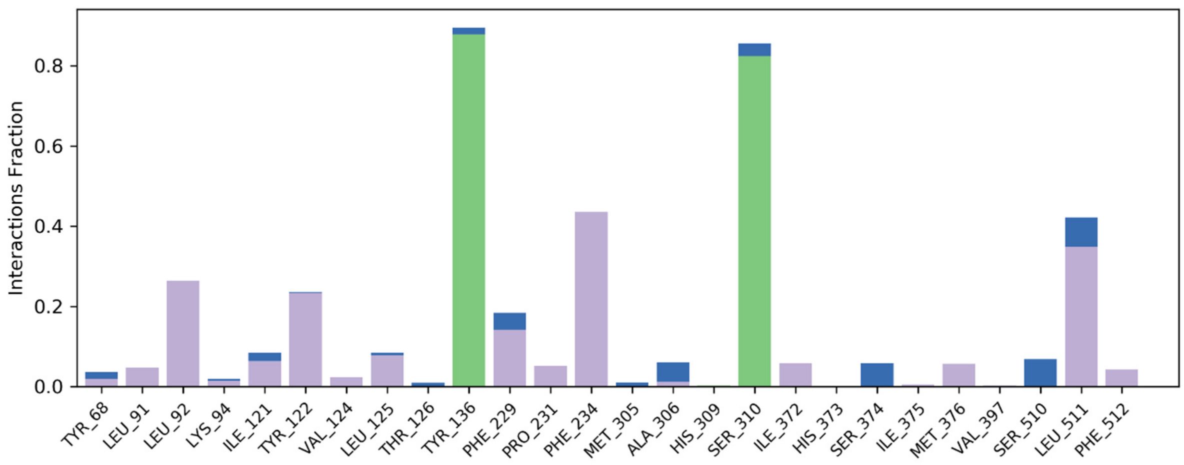

Supplementary Figure S7: Interactions of the *Madurella mycetomatis* CYP51 homology model residues with vilanterol during the entire MD simulations run. Different interactions were observed: hydrogen bonds (green); water bridges (blue) and hydrophobic interactions (grey).

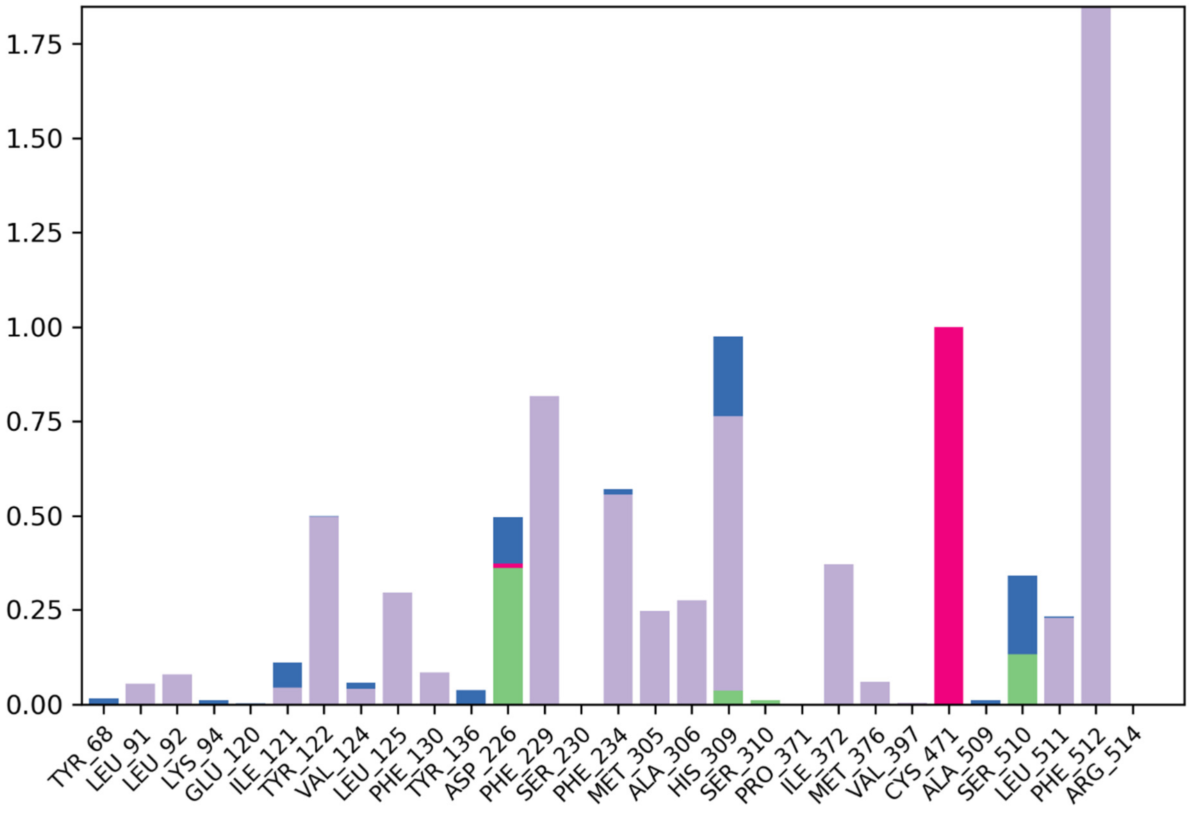

Supplementary Figure S8: Interactions of the *Madurella mycetomatis* CYP51 homology model residues with itraconazole during the entire MD simulations run. Different interactions were observed: hydrogen bonds (green); water bridges (blue), hydrophobic interactions (grey); and ionic interactions (red).

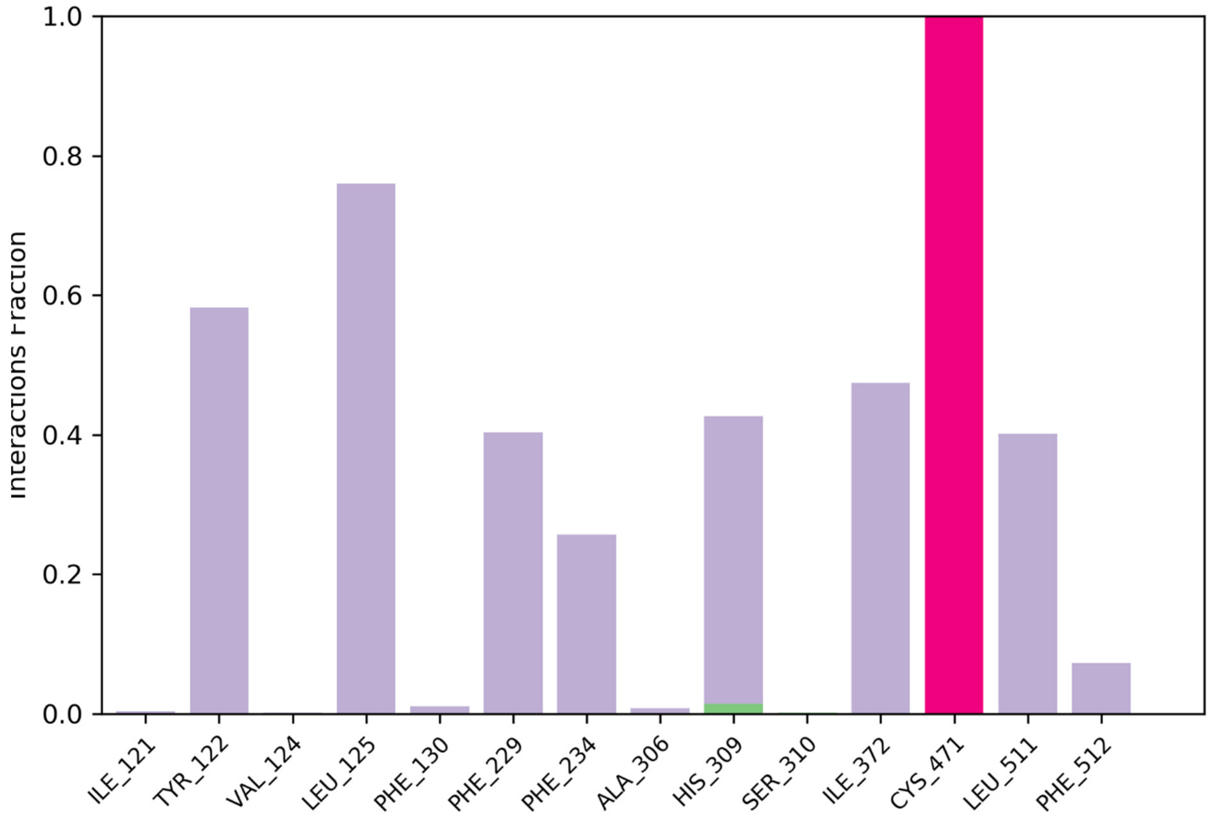

Supplementary Figure S9: Interactions of the *Madurella mycetomatis* CYP51 homology model residues with ravuconazole during the entire MD simulations run. Different interactions were observed: hydrogen bonds (green); hydrophobic interactions (grey); and ionic interactions (red).
